# Newborn metabolomic signatures of maternal per- and polyfluoroalkyl substance exposure and reduced length of gestation

**DOI:** 10.1038/s41467-023-38710-3

**Published:** 2023-05-30

**Authors:** Kaitlin R. Taibl, Anne L. Dunlop, Dana Boyd Barr, Yuan-Yuan Li, Stephanie M. Eick, Kurunthachalam Kannan, P. Barry Ryan, Madison Schroder, Blake Rushing, Timothy Fennell, Che-Jung Chang, Youran Tan, Carmen J. Marsit, Dean P. Jones, Donghai Liang

**Affiliations:** 1 Gangarosa Department of Environmental Health, Rollins School of Public Health, Emory University, Atlanta, GA USA; 2 Department of Gynecology and Obstetrics, School of Medicine, Emory University, Atlanta, GA USA; 3 Metabolomics and Exposome Laboratory, Nutrition Research Institute, University of North Carolina, Chapel Hill, NC USA; 4 Department of Pediatrics, New York University School of Medicine, New York, NY USA; 5 Department of Environmental Medicine, New York University School of Medicine, New York, NY USA; 6 Analytical Chemistry and Pharmaceuticals, RTI International, Research Triangle Park, Durham, NC USA; 7 Division of Pulmonary, Allergy, and Critical Care Medicine, Department of Medicine, School of Medicine, Emory University, Atlanta, GA USA

**Keywords:** High-throughput screening, Environmental impact, Biomarkers, Epidemiology

## Abstract

Marginalized populations experience disproportionate rates of preterm birth and early term birth. Exposure to per- and polyfluoroalkyl substances (PFAS) has been reported to reduce length of gestation, but the underlying mechanisms are unknown. In the present study, we characterized the molecular signatures of prenatal PFAS exposure and gestational age at birth outcomes in the newborn dried blood spot metabolome among 267 African American dyads in Atlanta, Georgia between 2016 and 2020. Pregnant people with higher serum perfluorooctanoic acid and perfluorohexane sulfonic acid concentrations had increased odds of an early birth. After false discovery rate correction, the effect of prenatal PFAS exposure on reduced length of gestation was associated with 8 metabolomic pathways and 52 metabolites in newborn dried blood spots, which suggested perturbed tissue neogenesis, neuroendocrine function, and redox homeostasis. These mechanisms explain how prenatal PFAS exposure gives rise to the leading cause of infant death in the United States.

## Abstract (web summary)

Mechanisms of the impact of PFAS (also known as forever chemicals) on adverse birth outcomes remain largely unknown. Here, authors identified tissue neogenesis, neuroendocrine function, and redox homeostasis as imprints of prenatal PFAS exposures and reduced gestational age in the newborn metabolome.

## Introduction

In 2020, there were an estimated 364,487 infants born preterm (22– < 37 completed gestational weeks) and 1,003,260 infants born early term (37–38 completed gestational weeks) in the United States (US)^1^. The annual rates of these adverse birth outcomes are consistently highest among Black Americans^1^. Preterm birth (PTB) and early term birth (ETB) are leading risk factors for morbidity and mortality during infancy, childhood, and early adulthood^2–5^. A reduced length of gestation is also linked to cardiovascular disease, diabetes, neurodevelopmental disorders, cancer, and other prevalent chronic health conditions across the life course^6–11^. The gestational age at birth is influenced by a complex interplay of psychosocial, behavioral, nutritional, and biological determinants, plus mounting evidence suggests environmental exposures potentiate the risk of PTB and ETB^12–16^. Recent work has demonstrated per- and polyfluoroalkyl substances (PFAS) are commonly present *in utero*, which may explain poor fetal growth and development^17–20^. However, most environmental epidemiologic studies have focused on white, highly educated populations and little is known about marginalized populations. Further, the underlying molecular mechanisms elicited by PFAS within the fetus’ metabolic, endocrine, and immune systems remain poorly understood.

PFAS are anthropogenic surfactants used by industries throughout the world and have a long history of use by the US Department of Defense^21^. Legacy PFAS, including perfluorooctanoic acid (PFOA), perfluorononanoic acid (PFNA), perfluorooctane sulfonic acid (PFOS), and perfluorohexane sulfonic acid (PFHxS), share in common a lipophobic carbon-fluorine chain, hydrophilic functional group, and proteinophilic attraction towards albumin and various fatty acid binding proteins^22–24^. These chemical properties result in long half-lives and foster persistence in the environment, which through a variety of exposure pathways lead to bioaccumulation in humans^25^.

In the US population, 99% of pregnant people have detectable levels of PFOA and PFOS in their blood^26^. Similarly, PFOA, PFOS, PFNA, and PFHxS are detected in nearly all maternal serum samples collected in the late first trimester or early second trimester in the Atlanta African American Maternal-Child Cohort^27^. During pregnancy, a proportion of the maternal PFAS body burden is able to cross the placental barrier into the uterus where the fetus is exposed ^28–31^. The direct effects of prenatal PFAS exposure on reduced length of gestation have been examined in several human populations^32–34^. However, the inconsistent and limited data among marginalized groups warrant more research, plus an unfulfilled public health priority is to hone the causal pathways for these exposure-outcome relationships. Our group and others have proposed interference with homeostatic processes from such environmental exposures *in utero* leads to a cascade of bioenergetic perturbation, endocrine disruption, and oxidative stress production, which may synergistically promote adverse birth outcomes^35–38^.

Untargeted metabolomics by high-resolution mass spectrometry combined with a *meet-in-the-middle* (MITM) analysis may help to characterize the molecular signatures of PFAS *in utero*, biological processes integral to fetal programming, and adverse phenotypes in early life^37^. Specifically, MITM allows for environmental exposures and health outcomes to be linked to metabolomic profiles, the global set of metabolites and systemic responses to internal doses of exogenous and endogenous substances^39,40^. Intermediate biomarkers and biological pathways for exposure-outcome relationships have been identified in several environmental epidemiologic studies using the MITM framework, including exposure to air pollution, tobacco smoke, and PFAS and fertility, PTB, and small-for-gestational age (SGA), respectively^37,41,42^. To our knowledge, no investigations have taken this approach to understand mechanistically how prenatal PFAS exposure influences the newborn metabolome and, in turn, how these responses are associated with gestational age at birth outcomes.

In a prospective birth cohort, we sought to profile the neonatal metabolome for molecular signatures of maternal PFAS concentrations during early to middle pregnancy and gestational age at birth outcomes among African American mother-newborn dyads in Atlanta, Georgia. Based on prior work, we hypothesized that prenatal PFAS exposure interferes with gestational length and fetal growth^37,43^. Additionally, we analyzed newborn dried blood spots (DBS), a minimally invasive biospecimen used for screening within 48 h of birth, with high-resolution metabolomics and the MITM framework to identify and measure the underlying metabolites and pathways.

Here, we show that an increase in maternal serum PFAS concentrations was prospectively associated with ETB and medically indicated early birth prior to full-term. The newborn DBS metabolome revealed perturbations in biological pathways involving amino acids, bioactive lipids, and enzymes, coenzymes, and cofactors underly the PFAS and gestational age at birth outcome relationships. We further characterized the molecular network by identifying salient metabolites in the newborn circulatory system, including L-DOPA, linoleic acid, and β-NAD.

## Results

### Study population characteristics

The characteristics of 267 African American pregnant people and newborns included in our study are summarized in Table 1. In early to middle pregnancy, the majority of mothers had a BMI considered overweight (*n* = 58; 22%) or with obesity (*n* = 109; 41%), were parous (*n* = 155; 58%), and did not use tobacco (*n* = 239; 90%) or marijuana (*n* = 177; 66%). At enrollment, the average participant age was 25.6 years (SD = 5.2) and 163 (61%) of the mothers were in the first trimester. Participants predominantly had a high school education or less (*n* = 153; 57%), public health insurance with Medicaid (*n* = 218; 82%), and an income level 132% or lower times that of the Federal Poverty Level (*n* = 153; 57%).

**Table 1:** Characteristics of 267 pregnant African American people and newborns in the Atlanta African American Maternal-Child cohort, 2016–2020

Characteristic	Participants, No. (%)^a^
*Delivery year*	
2016	44 (16)
2017	95 (36)
2018	64 (24)
2019	33 (12)
2020	31 (12)
Maternal age, mean (SD), y	25.6 ± 5.2
*Education*	
Less than high school	38 (14)
High school	115 (43)
Some college	68 (25)
College graduate or above	46 (17)
*Income-poverty ratio*^b^	
<100%	113 (42)
100 – 132%	40 (15)
133 – 149%	22 (8)
150 – 199%	49 (18)
200 – 299%	13 (5)
300 – 399%	11 (4)
≥400%	19 (7)
*Married or cohabitating*	
Yes	114 (43)
No	153 (57)
*Health insurance*	
Private	49 (18)
Public	218 (82)
*Hospital*	
Emory University Hospital (private)	103 (39)
Grady Hospital (public)	164 (61)
Parity	1.1 ± 1.2
Nulliparous	112 (42)
Primiparous	72 (27)
Multiparous	83 (31)
Prenatal BMI, kg/m^2 c^	29.0 ± 7.6
Underweight	8 (3)
Normal weight	92 (34)
Overweight	58 (22)
Obesity	109 (41)
*Marijuana use one month before pregnancy*	
Yes	90 (34)
No	177 (66)
*Tobacco use one month before pregnancy*	
Yes	28 (10)
No	239 (90)
Trimester serum sample collected^d^	11.3 ± 2.2
1st trimester	163 (61)
2nd trimester	104 (39)
*Delivery mode*	
Vaginal	120 (45)
Cesarean section	31 (12)
Missing	116 (43)
*Neonatal sex*	
Female	139 (52)
Male	128 (48)
*Gestational age at birth*^*e*^	
Preterm	31 (12)
Early term	82 (31)
Full-term	154 (57)
Gestational age at birth, mean (SD), weeks	38.7 (2.0)
*Labor and delivery course for early births*^*f*^	
Spontaneous	82 (69)
Medically indicated	31 (26)

*y* year, *BMI* body mass index (calculated as weight in kilograms divided by height in meters squared), *w* week.

^a^Reported percentages are composition ratios of each horizontal characteristic.

^b^Income-poverty ratio calculated as total family income divided by the Federal poverty threshold.

^c^BMI categorized as follows: underweight, <18.5 kg/m^2^; normal weight, 18.5 – 24.9 kg/m^2^; overweight, 25.0 – 29.9 kg/m^2^; and obesity, ≥30 kg/m^2^.

^d^Trimesters categorized as follows: 1^st^ trimester, 6 – 12 gestational weeks; 2^nd^ trimester, 13 – 17 gestational weeks.

^e^Gestational age at birth categorized as follows: preterm, 22 – <37 gestational weeks; early term, 37 – 38 gestational weeks; full-term, ≥39 gestational weeks.

^f^Reported percentages for labor and delivery course are composition ratios of preterm birth and early term birth versus 118 healthy full-term births.

There were 139 (52%) newborns assigned female sex at birth. The average gestational age at delivery was 38.7 weeks (SD = 2.0), with a total of 118 (51%) healthy and full-term, 31 (12%) preterm, and 82 (31%) early term. Among the early births (PTB or ETB) prior to full-term, 82 (69%) followed spontaneous labor and 31 (26%) followed medically indicated induction or C-section (Fig. S2).

All four PFAS were detected in 98–100% of maternal serum samples collected during early to middle pregnancy (Table S1). The GM (GSD) concentrations of PFOS were highest 1.43 ng/mL (2.72), followed by PFHxS 1.09 (2.30), then PFOA 0.57 (2.31), and lastly PFNA 0.25 (2.26). The log_2_-transformed PFAS concentrations (ng/mL) were weakly to moderately correlated with each other (Pearson $\rho$ range = 0.23–0.64) (Table S2).

### Prenatal PFAS in maternal serum and neonatal birth outcomes

**Fig. 1:**
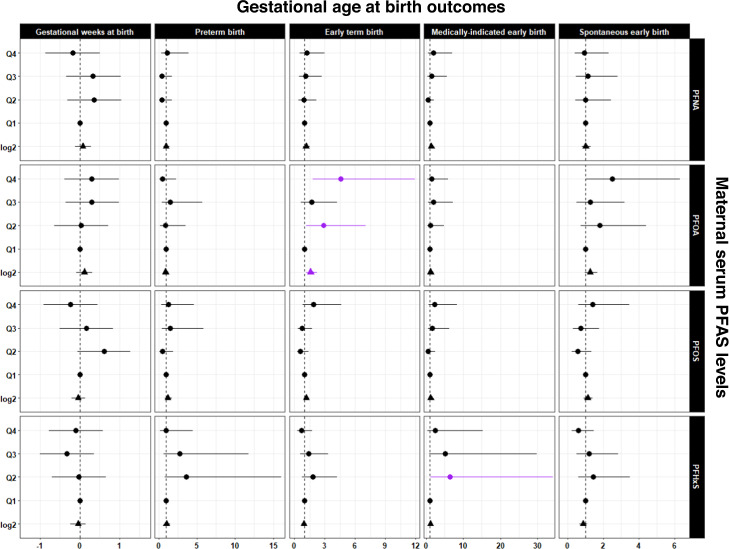
Dot-and-whisker plots showing the associations between prenatal serum PFAS levels and gestational age at birth outcomes among African American mother-newborn dyads in Atlanta, 2016–2020. Statistical tests were performed with two-sided multivariable linear or logistic regression with a significance level of p-value < 0.05. The sample size of independent dyads was as follows: *N* = 267 for gestational age at birth, *N* = 200 for early term birth and spontaneous early birth, *N* = 149 for preterm birth and medically indicated early birth. Data are presented as coefficient estimates (β) $\pm$95% confidence intervals (CI) or odds ratios (ORs) $\pm$95% confidence intervals (CI). The coefficient estimates (β) for gestational age and ORs for preterm birth, early term birth, medically indicated early birth (preterm birth or early term birth), and spontaneous early birth (preterm birth or early term birth) are on the *X*-axis. For the binary birth outcomes, the reference group was healthy full-term births. The vertical gray dashed line is the null value. Exposure to PFAS for every log_2_-unit increase and categorized by quartiles are on the Y-axis. Dots represent quartile exposures and triangles represent continuous exposures. Dots or triangles and whiskers color coded as purple are statistically significant at *p*-value < 0.05. The quartile cutoffs for PFOA (ng/mL) were as follows: Q1: <LOD – 0.42, Q2: 0.42 – 0.63, Q3: 0.63 – 0.96, Q4: 0.96 – 3.42. The quartile cutoffs for PFNA (ng/mL) were as follows: Q1: <LOD – 0.16, Q2: 0.16 – 0.28, Q3: 0.28 – 0.46, Q4: 0.46 – 1.51. The quartile cutoffs for PFOS (ng/mL) were as follows: Q1: <LOD – 1.04, Q2: 1.04 – 1.64, Q3: 1.64 – 2.46, Q4: 2.46 – 9.59. The quartile cutoffs for PFHxS (ng/mL) were as follows: Q1: <LOD – 0.66, Q2: 0.66 – 1.07, Q3: 1.07 – 1.93, Q4: 1.93 – 6.17. Note: Exact *p*-values are provided in Supplemental Table 3. PFAS, perfluoroalkyl substances; PFHxS, perfluorohexane sulfonic acid; PFOS, perfluorooctane sulfonic acid; PFOA, perfluorooctanoic acid; PFNA, perfluorononanoic acid; Q, quartile. Source data are provided as a Source Data file.

The effects of maternal PFAS concentration on gestational age at birth (continuous and categorical gestational weeks) and labor and delivery (spontaneous or medically indicated) outcomes relative to healthy, full-term birth are presented in Fig. 1 and Table S3. For every log_2_-unit increase in PFOA concentrations, the odds of ETB were 1.59 (95% CI: 1.15, 2.21) compared to healthy full-term birth. The odds ratio (OR) of ETB was also significantly increased among those in the 2nd quartile (OR = 2.85; 95% CI 1.16, 7.02) and 4th quartile (OR = 4.59; 95% CI 1.78, 11.89) of PFOA concentration versus the referent. Concentrations of PFOA categorized as the 3rd quartile increased the odds of ETB, but did not reach statistical significance (*p* > 0.05). Quartiles of PFHxS concentrations demonstrated a similar dose-response relationship with PTB and medically indicated early birth. Log_2_-transformed and quartile PFOA concentrations were associated with moderate increases in the odds of spontaneous labor. Finally, gestational age at delivery was inconsistently associated with PFAS concentrations.

### Neonatal metabolites, prenatal PFAS in maternal serum, and birth outcomes

We successfully obtained 6981 signals in the untargeted metabolomics dataset after data preprocessing, quality control procedures, and data filtering (Fig. S3). To increase the rigor of our study, we selected the MWAS with ≥100 signals enriched at the most conservative significance threshold for the remaining analyses, including pathway enrichment and molecular phenotyping using the MITM framework. In the PFAS MWAS, there were 435, 559, 154, and 1262 signals significantly associated with prenatal serum concentrations of PFOA (raw *p*-value < 0.05), PFOS (raw *p*-value < 0.05), PFNA (Bonferroni *q*-value < 0.01), and PFHxS (Bonferroni *q*-value < 0.01), respectively (Table S4). In the gestational age at birth outcomes MWAS, there were 318, 199, 244, 669, and 142 signals significantly associated with medically indicated early birth (FDR *q*-value < 0.20), ETB (FDR *q*-value < 0.20), spontaneous early birth (FDR *q*-value < 0.05), PTB (FDR *q*-value < 0.05), and gestational weeks (Bonferroni *q*-value < 0.01), respectively (Table S5). The number of significantly enriched signals associated with PFAS concentrations did not materially change when gestational week at sample collection was removed from the models (Table S6).

**Fig. 2:**
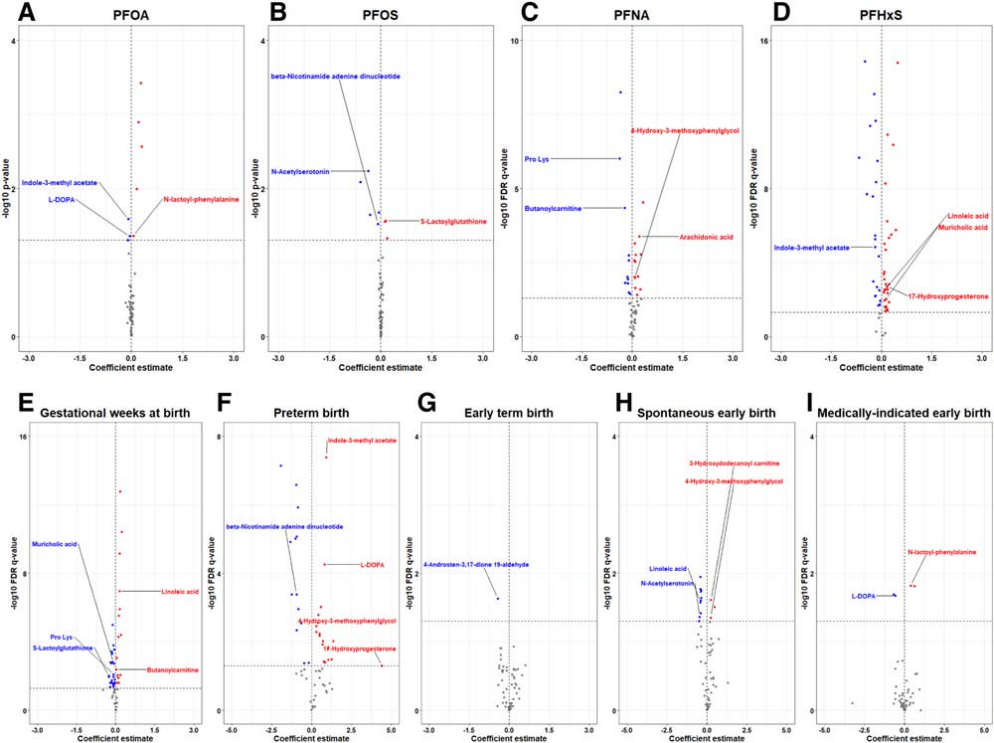
Volcano plots of identified metabolites associated with prenatal serum PFAS levels and gestational age at birth outcomes in Atlanta, 2016 – 2020. Blue dots represent metabolites with negative associations, red dots represent metabolites with positive associations, and gray dots represent metabolites with non-significant associations among those confirmed and annotated. The coefficient estimate (β) from the MWAS is on the *X*-axis. The significance threshold is on the *Y*-axis. The vertical gray dashed line represents the null value. Select metabolites are annotated on the plots. **A** Volcano plot of metabolites associated with PFOA. Statistical tests were performed with two-sided multivariable linear regression. There were *N* = 267 independent dyads in the analytic sample. No adjustments were made for multiple comparisons. The horizontal gray dashed line represents the log_10_ *p*-value = 0.05. **B** Volcano plot of metabolites associated with PFOS. Statistical tests were performed with two-sided multivariable linear regression. There were *N* = 267 independent dyads in the analytic sample. No adjustments were made for multiple comparisons. The horizontal gray dashed line represents the log_10_ *p*-value = 0.05. **C** Volcano plot of metabolites associated with PFNA. Statistical tests were performed with two-sided multivariable linear regression. There were *N* = 267 independent dyads in the analytic sample. FDR from multiple comparisons was corrected with the Benjamini–Hochberg procedure. The horizontal gray dashed line represents the log_10_ FDR *q*-value = 0.05. **D** Volcano plot of metabolites associated with PFHxS. Statistical tests were performed with two-sided multivariable linear regression. There were *N* = 267 independent dyads in the analytic sample. FDR from multiple comparisons was corrected with the Benjamini–Hochberg procedure. The horizontal gray dashed line represents the log_10_ FDR *q*-value = 0.05. **E** Volcano plot of metabolites associated with gestational weeks at birth. Statistical tests were performed with two-sided multivariable linear regression. The reference group was healthy full-term births. There were *N* = 267 independent dyads in the analytic sample. FDR from multiple comparisons was corrected with the Benjamini-Hochberg procedure. The horizontal gray dashed line represents the log_10_ FDR *q*-value = 0.05. **F** Volcano plot of metabolites associated with preterm birth. Statistical tests were performed with two-sided multivariable logistic regression. The reference group was healthy full-term births. There were *N* = 149 independent dyads in the analytic sample. FDR from multiple comparisons was corrected with the Benjamini–Hochberg procedure. The horizontal gray dashed line represents the log_10_ FDR *q*-value = 0.05. **G** Volcano plot of metabolites associated with early term birth. Statistical tests were performed with two-sided multivariable logistic regression. The reference group was healthy full-term births. There were *N* = 200 independent dyads in the analytic sample. FDR from multiple comparisons was corrected with the Benjamini-Hochberg procedure. The horizontal gray dashed line represents the log_10_ FDR *q*-value = 0.05. **H** Volcano plot of metabolites associated with spontaneous early birth. Statistical tests were performed with two-sided multivariable logistic regression. The reference group was healthy full-term births. There were *N* = 200 independent dyads in the analytic sample. FDR from multiple comparisons was corrected with the Benjamini-Hochberg procedure. The horizontal gray dashed line represents the log_10_ FDR *q*-value = 0.05. **I** Volcano plot of metabolites associated with medically indicated early birth. Statistical tests were performed with two-sided multivariable logistic regression. The reference group was healthy full-term births. There were *N* = 149 independent dyads in the analytic sample. FDR from multiple comparisons was corrected with the Benjamini-Hochberg procedure. The horizontal gray dashed line represents the log_10_ FDR *q*-value = 0.05. MWAS, metabolome-wide association study; PFAS, per- and polyfluoroalkyl substances; PFHxS, perfluorohexane sulfonic acid; PFOS, perfluorooctane sulfonic acid; PFOA, perfluorooctanoic acid; PFNA, perfluorononanoic acid; FDR, false discovery rate. Source data are provided as a Source Data file.

**Fig. 3:**
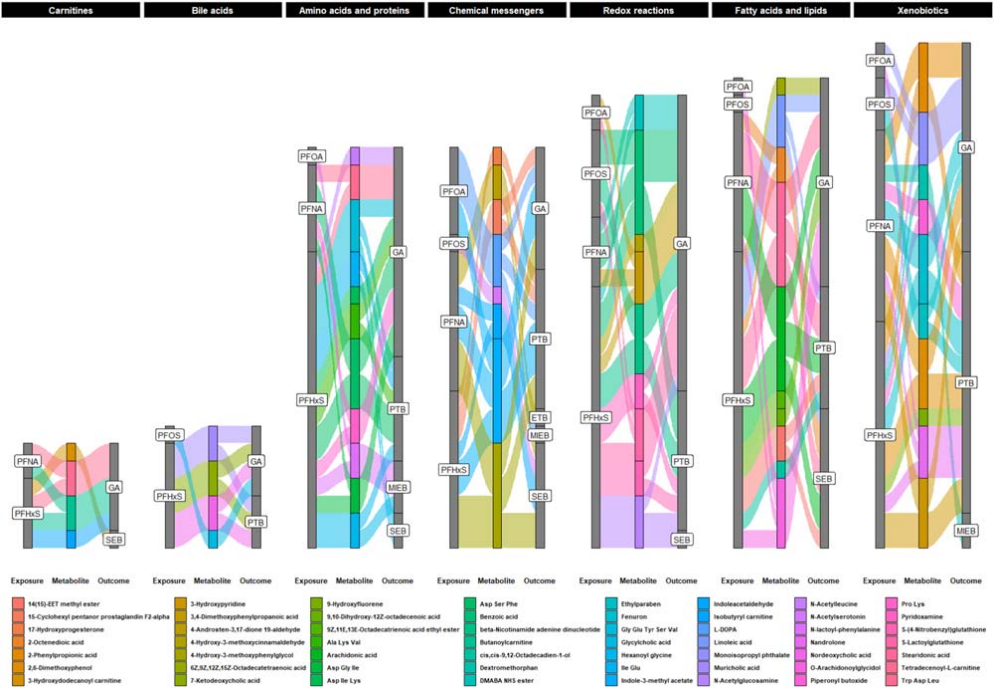
Alluvial plots of identified metabolites that relate the association of prenatal serum PFAS levels with gestational age at birth outcomes in Atlanta, 2016–2020. The sample size of independent dyads was as follows: *N* = 267 for gestational age at birth, *N* = 200 for early term birth and spontaneous early birth, *N* = 149 for preterm birth and medically indicated early birth. The gray stacked bars indicate the frequency and relative proportion of PFAS exposure or gestational age at birth outcomes associated with the metabolite. The colored stacked bars indicate the frequency and relative proportion of metabolites associated with PFAS exposure and gestational age at birth outcomes. The legend shows which color corresponds to one of the 56 metabolites. Abbreviations**:** PFAS, perfluoroalkyl substances; PFHxS, perfluorohexane sulfonic acid; PFOS, perfluorooctane sulfonic acid; PFOA, perfluorooctanoic acid; PFNA, perfluorononanoic acid; PTB, preterm birth; ETB, early term birth; MIEB, medically indicated early birth; SEB, spontaneous early birth; GA, gestational weeks at birth. Source data are provided as a Source Data file.

In total, we found 664 overlapping signals in one or more of the PFAS MWAS and one or more of the gestational age at birth outcomes MWAS when the significance threshold was set to *p*-value < 0.05 for PFOA and PFOS and FDR < 0.05 for all others (Table S7). Upon signal identification and annotation, a total of 56 overlapping metabolites were matched with high confidence ontology levels, including nine labeled as OL1, 12 labeled as OL2a, 10 labeled as OL2b, and 25 labeled as PDa (Table S8 and Table S9). The metabolite profile consisted of carnitines, bile acids, amino acids and proteins, chemical messengers, redox reactions, fatty acids and lipids, and xenobiotics (Figs. 2 and  3). Amino acids and proteins were the largest of these classes with 11 metabolites, which were predominantly associated with maternal serum PFNA and PFHxS levels plus gestational age and PTB. Carnitines and bile acids overlapped with the fewest number of PFAS exposures and gestational age at birth outcomes whereas chemical messengers overlapped across all nine MWAS. The polyunsaturated ω-6 fatty acids, arachidonic acid and linoleic acid, as well as tetradecenoyl-L-carnitine, which shuttles saturated long-chain fatty acids to mitochondria, were top metabolites and shared in common an association with PFHxS and gestational weeks (Fig. 2C, D, E, H). Another group of metabolites with a high frequency of overlap included indole-3-methyl acetate, 4-hydroxy-3-methyoxyphenylglycol, and β-nicotinamide adenine dinucleotide (β-NAD) (Fig. 2A–D, F, H).

### Neonatal pathways, prenatal PFAS in maternal serum, and birth outcomes

**Fig. 4:**
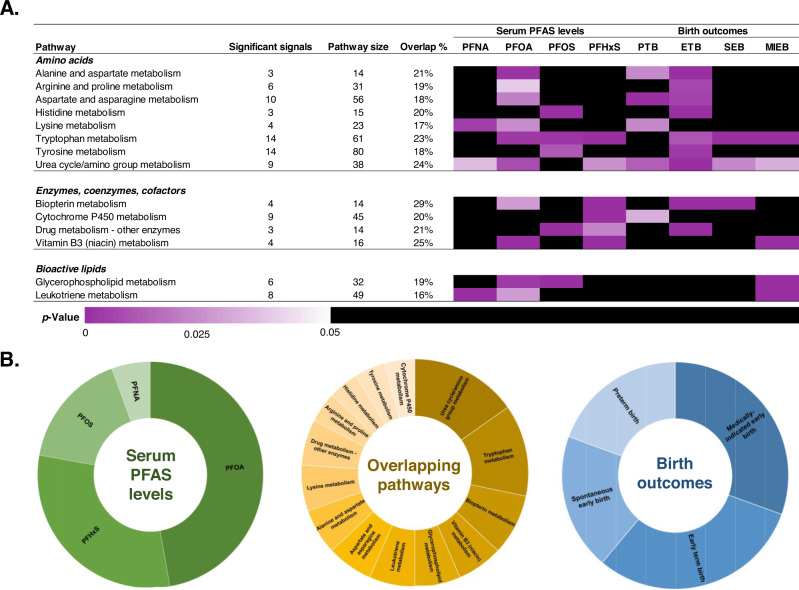
Enriched pathways that relate the association of prenatal serum PFAS levels with gestational age at birth outcomes in Atlanta, 2016–2020. The sample size of independent dyads was as follows: *N* = 200 for early term birth and spontaneous early birth, *N* = 149 for preterm birth and medically indicated early birth. **A** Heat map of the γ-adjusted *p*-values. A γ *p*-value is calculated based on permutations that randomly resamples the list of total features for a number of features equal to the significant set many times to create a γ null distribution. Cell color corresponds to the p-value for a metabolic pathway that overlaps in at least one PFAS metabolome-wide association study (MWAS) and gestational age at birth outcome MWAS. The reference group was healthy full-term births for the following MWAS: preterm birth, early term birth, medically indicated early birth (PTB or ETB), and spontaneous early birth (PTB or ETB). The significant signals are the average numbers of significant putative metabolites enriched in the overlapping pathways and associated an exposure or outcome. The pathway size is the average number of significant putative metabolites in the overlapping metabolic pathways. Overlap % is the relative proportion of significant signals to the pathway size. The metabolic pathways are grouped by amino acids, enzymes, coenzymes, and cofactors, and bioactive lipids. **B** Sunburst charts showing the frequency of overlap for the serum PFAS levels (green), overlapping pathways (gold), and gestational age at birth outcomes (blue). The largest sections indicate the greatest amount of overlap and the smallest sections indicate the least amount of overlap. Abbreviations**:** PFAS, per- and polyfluoroalkyl substances; PFHxS, perfluorohexane sulfonic acid; PFOS, perfluorooctane sulfonic acid; PFOA, perfluorooctanoic acid; PFNA, perfluorononanoic acid; PTB, preterm birth; ETB, early term birth; MIEB, medically indicated early birth; SEB, spontaneous early birth; GA, gestational weeks at birth. Source data are provided as a Source Data file.

We identified 14 unique, overlapping pathways associated with PFAS exposure and gestational age at birth outcomes, which were broadly related to micronutrients, bioenergetics, bioactive lipids, and enzymes and cofactors (Fig. 4 and Table S10). Gestational weeks at birth had no pathways that were present in the PFAS pathway enrichment analyses after removing those with <10% overlap. PFOS and PFHxS, containing a sulfonic acid moiety, shared in common the pathway for drug metabolism with ETB. PFOA and PFNA, containing a carboxylic acid moiety, shared in common lysine metabolism with PTB and leukotriene metabolism with medically indicated early birth. Glycerophospholipid metabolism overlapped with PFOA and PFOS and medically indicated early birth. The metabolic pathway for amino acids in the urea cycle was significantly enriched in all pathway enrichment analyses performed, except for PFOS. Tryptophan metabolism was significantly enriched in seven out of the nine pathway enrichment analyses, excluding PFNA and PTB. Lastly, across the nine pathway enrichment analyses performed, the percent of the proportion of significant putative metabolic signals versus the overall pathway size ranged from 10% to 64%.

The pathways associated with PFAS exposure and gestational age at birth outcomes were generally consistent across the enrichment analyses performed at serially reduced significance thresholds (Table S11 and Table S12). Additionally, the overlapping pathways significantly enriched in the PFAS sensitivity analyses did not greatly differ from the results obtained in the main analyses (Table S13).

## Discussion

**Fig. 5:**
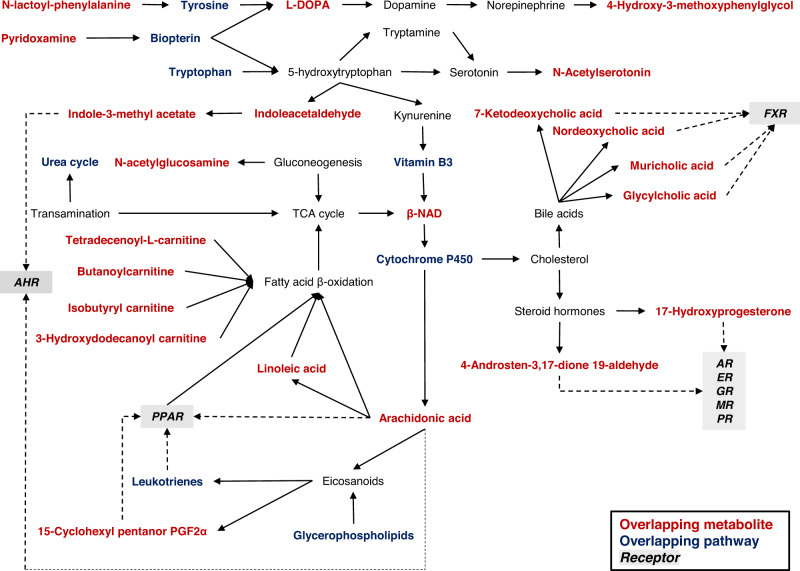
Proposed molecular network of mechanisms and biomarkers in the newborn dried blood spot metabolome underlying the association of prenatal serum PFAS levels with gestational age at birth outcomes in Atlanta, 2016–2020. Abbreviations**:** PFAS, per- and polyfluoroalkyl substances; AHR, aryl hydrocarbon receptor; PPAR, peroxisome proliferator-activated receptor; AR, androgen receptor; ER, estrogen receptor; GR, glucocorticoid receptor; MR, mineralocorticoid receptor; PR, progestin receptor; FXR, farnesoid X receptor; TCA cycle, tricarboxylic acid; L-DOPA, levodopa; β-NAD, β-nicotinamide adenine dinucleotide; 15-cyclohexyl pentanor PGF2α, 15-cyclohexyl pentanor prostaglandin F2α. Source data are provided as a Source Data file.

To our knowledge, this is the first study that has used newborn DBS metabolomics to explore the molecular mechanisms between prenatal PFAS exposure and gestational age at birth outcomes. We found maternal serum PFAS levels during early to middle pregnancy, which were surrogate measurements for fetal exposure to PFAS, were associated with early birth (PTB or ETB) prior to full-term among African American pregnant people and their newborns. Furthermore, the neonatal metabolome had an intermediate role for this relationship and offered insights into the underlying molecular network, as presented in Fig. 5. The biological pathways and biomarkers indicated perturbations in tissue neogenesis, neuroendocrine function, and redox homeostasis, which may have long-term health consequences beyond time of birth.

In comparing pregnant people in the Atlanta African American Maternal-Child Cohort versus participants matched on sex, age, and race in NHANES, the detection rate and geometric average of PFOA, PFOS, and PFNA are similar while PFHxS is significantly higher in our study population^27^. Relative to other prospective cohorts in the US, maternal serum PFAS concentrations collected during early pregnancy are comparable to those observed in Chemicals In Our Bodies and Illinois Kids Infant Development Study, and lower than those observed in Project Viva, LIFECODES, and Healthy Start Study, which have a majority white and higher SES study population^44–47^.

While a growing body of evidence suggests exposure to PFAS increase the risk of PTB, as supported by our findings, other studies linking spontaneous or medically indicated early birth in relation to serum PFAS levels have proven inconclusive^34,48–51,32,52^. Similarly, the relationship between various PFAS measured in pregnant people and gestational age of newborns has been difficult to replicate across environmental epidemiologic studies^20,53,54^. A potential explanation for these observed differences is the proportion of PTB and ETB were higher in the present study relative to prior work, which mirrors the current trends of maternal and child health outcomes among African American families. Our findings provide credence to a relationship between prenatal PFAS exposure and adverse birth outcomes in populations routinely excluded from environmental epidemiologic studies.

When compared to adults, the developing fetus is exposed to a higher dose of environmental chemicals due to immature size and has insufficient enzymatic capacity to protect against xenobiotics^55^. Our analyses suggest maternal PFAS levels may affect fetal P450 and other drug-metabolizing enzymes, as made evident in the newborn DBS metabolome, and are associated with PTB and ETB, respectively. Such exposures may interfere with developmental transitions in CYP expression, which occur throughout gestation and postnatally, thereby influencing the disposition of PFAS^56^. Since mitochondrial P450 catabolize cholesterol, these enzymatic mechanisms may also point to perturbed steroidogenesis, bile acid synthesis, and vitamin D3 activation, which our group and others have shown to be affected by PFAS^57,58,37,59–67^.

In early life and beyond, bioenergetic processes rely on vitamin B3 to sustain the reduced nicotinamide adenine dinucleotide phosphate (NADPH) pool, which is involved in mitochondrial oxidative phosphorylation, redox homeostasis, and protein synthesis and function^68,69^. We found maternal serum PFOA and PFHxS levels affected neonatal vitamin B3 metabolism, which was associated with medically indicated early birth prior to full-term. Additionally, the vitamin B3 metabolite β-NAD was attenuated by PFOS and PFHxS. Taken altogether, a reduced length of gestation may arise in part from prenatal PFAS exposure targeting vital pathways for energy metabolism.

Biopterins are cofactors for the biosynthesis of neurotransmitters, hormone precursors, and vasodilators plus indicate oxidative stress since reactive oxygen species (ROS) limit their bioavailability^70–74^. PFAS generate an excessive amount of ROS in vitro and deplete antioxidant reserves in vitro and in vivo^75–78^. We found several antioxidative metabolites, including benzoic acid and S-lactyolglutathione, were associated with maternal serum PFHxS concentrations and gestational age, which may be in response to exposure. In addition, oxidative stress biomarkers have been linked to an increased risk of preterm birth^79^. Our metabolomic findings need to be validated in other fetal and neonatal populations, but illustrate the potential for oxidative stress to serve as a shared link between prenatal PFAS exposure and reduced length of gestation.

We observed most of the intermediate metabolomic signatures between PFAS in pregnant people and gestational age at birth outcomes were related to amino acids, consistent with our prior work using maternal serum metabolomics^37^. Complex systems of amino acid transporters in the placenta tightly regulate the flux of amino acids between mother and fetus to ensure the demand for tissue neogenesis is met and so byproducts of protein metabolism are removed^80,81^. It has been shown that PFAS exposure during gestation reduces amino acid transporter expression and system activity, which subsequently limits fetal growth and development. For example, there is reduced expression of SNAT4, an isoform of the system A amino acid transporter, in the placentas of pregnant mice exposed to PFOS^82^. In the future, these early life mechanisms of amino acid metabolism may serve as therapeutic targets for reduced length of gestation caused by PFAS *in utero*.

Prenatal exposure to PFAS may potentiate risk of PTB and ETB by disrupting the metabolism of tryptophan and tyrosine. Tryptophan results in serotonin, kynurenine, tryptamine, and indole production, which take part in neurological, hormonal, and immunological processes during pregnancy and afterwards^83–85^. Perturbations to this amino acid during pregnancy have been implicated in chronic inflammation disease states among mothers and adverse neurodevelopmental outcomes among offspring^86^. Similarly, tyrosine has a prominent role in the dopaminergic system of the placenta-brain-axis. Tyrosine hydroxylase synthesizes the dopamine precursor, levodopa (L-DOPA), from a combination of tyrosine, oxygen, and biopterin to support fetal neurodevelopment and survival^72,87^. Our results suggest the indirect effects of PFAS on reduced length of gestation may occur through neuroendocrine pathways, and follow-up research should prioritize the investigation of related chemical messengers among premature and early newborns.

The newborn DBS metabolome was enriched with metabolic pathways for glycerophospholipids and leukotrienes along with arachidonic acid, linoleic acid, and 15-cyclohexyl pentanor prostaglandin F2α metabolites. In other populations with occupational, community, and background exposure, dyslipidemia is commonly present regardless of age, possibly suggesting PFAS affect a lipid pathway conserved across the life course^60,88–92^. Specific to fetal growth and development, perturbed bioactive lipids may contribute to the mechanisms underlying the relationship between PFAS toxicity *in utero* and reduced length of gestation.

The relationship between serum PFAS levels and lipids has been primarily attributed to suppressed mitochondrial fatty acid β-oxidation, attenuated farsenoid x receptor (FXR) via bile acids, and activated peroxisome proliferator-activated receptors (PPAR)^61,93,94^. These three processes have the potential to drive bioenergetic perturbation and oxidative stress production during gestation, which may be antecedent molecular events to adverse birth outcomes. One proposed mechanism is upregulated PPAR signaling from PFAS exposure alters arachidonic acid metabolism and its downstream metabolites, which leads to oxidative stress and inflammation, based on experimental work in rodents using metabolomics, proteomics, and transcriptomics^95–98^. Pregnancy may represent a sensitive window for PFAS to interact with eicosanoids and their precursors because of their role in the onset of labor, which has long been recognized by clinicians^99^. The application of targeted methods is needed to validate and expand upon the results presented here, particularly the roles of bioactive lipids and inflammatory processes that precede or concomitantly occur with a reduced length of gestation.

The exact role of prenatal PFAS exposure, as well as their overall significance, in driving maternal and child health disparities among African Americans remains unclear. Perhaps, exposure to PFAS compounds the physiological effects of gendered racial stress and discrimination during pregnancy, ultimately leading to an early birth. In consideration of the mechanisms identified here, more research is needed to understand the potential joint effects of environmental chemicals and non-chemical stressors on perinatal health outcomes.

A combination of systems biology methods and tools, sampling techniques, and biostatistical approaches strengthened our study. First, we leveraged an untargeted high-resolution metabolomics platform with the MITM framework, which together positioned us to identify and measure the links among exposures, molecular signatures, and outcomes. Our group initially examined maternal metabolomic perturbations associated with prenatal PFAS exposure and fetal growth outcomes in the Atlanta African American Maternal-Child Cohort^37^. As a step to validate and expand upon such findings, we used newborn DBS samples to make inferences about how the fetal biological system is affected by PFAS exposure *in utero*, a major advancement over studies that have relied on cord blood. Our study also serves as a proof-of-concept for integrating high-throughput -omics, newborn screening protocols, and environmental molecular epidemiology, which will be increasingly important as public health and translational science advance toward a precision medicine model. Third, our phenotypic classification of gestational age at birth outcomes was based on medical record abstraction by medical personnel, minimizing the chance of misclassification that might affect studies based on self-report. Our research protocol also considered the phenotypic classification of births as early (PTB or ETB) or full-term based on early pregnancy dating with further phenotypic classification of early births following spontaneous labor or medically indicated labor, allowing us to distinguish these different pathways to early birth in our analyses. There is a recognized need for further research into factors increasing the risk of ETB as this is an understudied birth outcome and infants born in this category disproportionately experience excess morbidity relative to full-term infants^100^. Lastly, our study population included exclusively African Americans, a population that is sorely underrepresented in the extant literature.

Our study also had several limitations. Only the individual effects of PFAS were included in this study, but mixture effects are plausible and alternative PFAS with lower detection rates and/or shorter half-lives may contribute to the observed associations. There is a potential for interaction of the studied PFAS with other environmental chemicals from co-exposure, which may be antagonistic, synergistic, or additive, and mask the true health effects of PFAS. Future population-based studies are needed to investigate environmental chemical mixtures and emerging PFAS, for which there are thousands, in both the intrauterine and extrauterine environments. Our limited sample size also necessitates a larger-scale study, which newborn DBS sampling could facilitate. There was a relatively small number of pregnant people who experienced PTB, which may explain why we did not observe a significant dose-response relationship when participants were categorized by exposure quartiles. Furthermore, the pathogenesis of adverse birth outcomes is likely multifactorial and influenced by genetics, socioeconomic status and behaviors, diet, environment, and notably for minority racial and ethnic groups, structural factors such as social inequality and systemic racism. As a result, we cannot rule out the possibility of residual confounding. By the same token, there is an ongoing discussion in environmental epidemiology about how maternal BMI is associated with serum PFAS concentrations during pregnancy. Based on the body of literature available today, we treated maternal BMI as a confounder, although more research is needed to clarify the direction of effect between adiposity and PFAS in pregnant populations^101,102^. The newborn DBS samples and panel of gestational age at birth outcomes were measured during the same time period, which hinders causal inference for the metabolomic signal-outcome associations. Specific to the newborn DBS samples collected via heel-stick, critics have raised concerns over potential uneven cell distribution, cell lysis, hematocrit variation, and pre-analytical clotting when analyzing this biomatrix using untargeted metabolomics^103–105^. Although the untargeted DBS metabolomics analysis was normalized by the size and weight of the DBS, we were unable to conduct additional, potentially more optimal normalization methods, including adjustment for hemoglobin, specific gravity, protein, or potassium due to limited volume of available sample^106^. Finally, there are fewer assumptions required for using the MITM framework compared to formal mediation analysis and the results should be interpreted with caution.

Taken altogether, our study provides novel evidence and supports the current mechanistic understanding of how PFAS *in utero* influence PTB and ETB risk. Additional research is needed to validate our findings in other populations and to investigate the potential of these metabolic mechanisms and biomarkers for detection, treatment, and prevention strategies in public health and clinical settings.

## Methods

### Study design and population

Participants from the Atlanta African American Maternal-Child Cohort (ATL AA hereafter) were included in the present analysis. This ongoing, prospective birth cohort enrolls pregnant African Americans between 6 and 17 weeks gestation at Emory Midtown Hospital and Grady Hospital, which serve socioeconomically diverse populations in Atlanta, Georgia, and extends dyad follow-up through age six. Additional information regarding the cohort profile and data collection is described in detail elsewhere^107,108^. Participants were eligible for inclusion if they self-identified as African American or Black, and were born in the US, between 18 and 40 years old, pregnant with a singleton, fluent in English, and had no chronic medical conditions. All participants provided written, informed consent to participate in the study, which was approved by the Institutional Review Board at Emory University (approval reference number 68441). Participant data are confidential and proprietary information to the ATL AA cohort.

A total of 279 mothers and newborns enrolled in the ATL AA cohort had serum and DBS samples, respectively. At the time of our analysis, there were 273 participants with complete data available for PFAS measurements and DBS metabolomics (Fig. S1). The live births occurred between June 2016 and June 2020. We excluded newborns with congenital anomalies (*n* = 6) from our analysis. The remaining 267 dyads were included in the PFAS metabolome-wide association studies (MWAS), gestational age MWAS, and prenatal PFAS-gestational age analysis.

### Covariates

A thorough review of the literature was completed a priori to identify additional covariates that potentially confound the relationships between our exposures and outcomes. In previous analyses conducted in the ATL AA Cohort, we found maternal body mass index (BMI), use of tobacco and marijuana, education, parity, and age are significant predictors of prenatal PFAS exposure^27^. Tobacco smoke is also associated with perturbations in the maternal serum metabolome and adverse birth outcomes^42^. Altogether, this information guided our construction of a directed acyclic graph (DAG; Fig. S2). Parity (0–8) and use of tobacco and marijuana one month prior to pregnancy (yes/no) were abstracted from prenatal medical records. Other social, economic, and demographic factors were obtained by questionnaire. The mothers self-reported age (years), highest attained educational level (less than high school, high school, some college, college and above), number of members in household and household income for determining income-to-poverty ratio (<100%, 100–150%, 150–300%, 300–399%, ≥400%), health insurance type (private/public), and partner status (married or cohabitating, unmarried or not cohabitating) at time of enrollment were collected. The mother’s BMI (kg/m^2^) was calculated from the first clinical visit’s weight and height measurements during early pregnancy and categorized as follows: underweight <18.5 kg/m^2^, normal weight 18.5– < 25 kg/m^2^, overweight 25– < 30 kg/m^2^, and with obesity ≥30 kg/m^2^. Since PFAS undergo trans-placental transfer throughout the course of pregnancy, the gestational week when the serum samples were collected from the mothers was included as a continuous covariate in the models which included PFAS as the exposures. Finally, neonatal sex was believed to affect the exposures and outcomes, and thus adjusted in the models.

### Quantification of perfluoroalkyl substances

Whole blood samples were collected from pregnant participants between 6 and 17 gestational weeks via venipuncture in an antecubital vein. These samples were transported to the National Institute of Environmental Health Sciences (NIEHS) Children’s Health Exposure Analysis Resource (CHEAR) laboratories, including Emory University’s Laboratory of Exposure Assessment and Development for Environmental Research (LEADER), New York University’s Laboratory Hub (NYU), and the Wadsworth Center Laboratory in New York. The samples were centrifuged (2000*g*) to obtain the serum fraction, which was used to quantify PFOA, PFNA, PFOS, and PFHxS, and subsequently stored at −80 °C. The analytical procedures and methods to quantify PFAS have been described in detail elsewhere^27,109^. Briefly, after randomization, the sera were spiked with isotopic internal standards followed by solid-phase extraction and analysis by high-performance liquid chromatography-tandem mass spectrometry (LC-MS/MS). Individual PFAS species were quantified by isotope dilution calibration.

The Human Health Exposure Analysis Resource (HHEAR) replaced CHEAR in 2019 and requires adherence to strict quality control procedures so services at any of the laboratories are harmonized and comparable to globally recognized, external laboratories^110–112^. Additionally, all of the laboratories participate in the German External Quality Assessment Scheme (https://app.g-equas.de/web/) to demonstrate data quality assurance in serum PFAS analysis.

### Birth outcomes

Gestational age at birth (weeks) was calculated from the estimated date of conception, based on the mother’s self-reported last menstrual period and/or early pregnancy ultrasound, to the date of delivery^113,114^. In accordance with the American College of Obstetricians and Gynecologists (ACOG) and the Society for Maternal Fetal Medicine (SMFM), gestational age at birth was categorized as follows: gestational age 22– < 37 weeks was considered PTB; gestational age 37–38 weeks was considered ETB; full-term births had a gestational age ≥39 weeks^100^. Information about the presence or absence of pregnancy complications and the labor and delivery course was abstracted from the medical record and used to further categorize the early births (those that were PTB or ETB) as spontaneous (i.e., following spontaneous labor or premature rupture of membranes) or medically indicated (i.e., following C-section or induction for a maternal or fetal indication); in addition, full-term births were further categorized as healthy full-term births if the pregnancy was without complications (such as gestational hypertension, gestational diabetes, or preeclampsia) and these healthy full-term births were used as the referent category in analyses.

### *Untargeted High-Resolution Metabolomics*

Newborn DBS samples are routinely collected at the time of birth for medical screening and public health surveillance then archived for future biomonitoring purposes. Metabolomics analysis of newborn DBS samples has recently emerged as a powerful method to phenotype exposures and health outcomes, such as those in the present study, at the population level^115–118^. In comparison to whole blood samples containing serum and plasma fractions, newborn DBS samples are also reliable, reproducible, and representative biospecimens of the circulating metabolome in humans, plus carry the advantage of novel insights into the intrauterine environment, which are largely uncharacterized^118^.

DBS samples were collected within 24–48 h after birth from a heel stick performed by trained hospital nursery personnel using a standardized protocol that involves cleaning the skin of the heel with 75% isopropanol (wiping off any excess alcohol and allowing the skin to air-dry), using a sterile 2.5 mm lancet to obtain the sample, and collecting the sample onto a standard Guthrie card by saturating each circle with ~75 uL of blood^119^. On the day of collection, card specimens were transported to the Georgia Department of Public Health Laboratory for storage in a walk-in refrigerator (2–8 °C) without a desiccant for up to three months and then transported to an Emory University Laboratory for storage in freezer (−80 °C) within gas-impermeable bags with dessicant until assay^119^. For this study, we obtained single 15-mm punches (equivalent to ~50 uL whole blood) that were collected from 279 children between 2016 and 2020^116^. An additional set of 15-mm punches was obtained from adjacent portions of filter paper from the same Guthrie cards to use as blanks.

Untargeted high-resolution metabolomics profiling was conducted in a single run by the North Carolina HHEAR Hub (NC HHEAR Hub) in UNC Nutrition Research Institute, North Carolina Research Campus (Kannapolis, NC) using established methods. Blood spot samples were extracted with 1 mL ice-cold methanol containing 500 ng/mL L-tryptophan-d5, vortexed at 5,000 rpm for 10 min, and then sonicated for another 20 min. After centrifugation at 16,000 rcf for 10 min at 4 °C, 600 µL of the extracted supernatant was transferred into a 2.0 mL low-bind microfuge tube. An additional 70 µL of the extracted supernatant from each of the study samples was pooled together and then redistributed into new set of tubes with 600 µL per tube to generate total study pool samples. The empty blood spot card (without blood) matched with each of the study samples in terms of size and material was used as a blank and processed using the same procedures as the study sample. Supernatant (70 µL) from each of the blanks were mixed together and then distributed into multiple low-bind 2.0 mL tubes with a volume of 600 µL per tube. All supernatant aliquots (600 µL) including study samples, study pools, and blanks were dried by vacuum concentrator overnight and reconstituted with 100 µL water-methanol (95:5, v/v) for the ultra-high performance (UHP) LC-HR-MS analysis. Aliquots of 50 µL of NIST reference plasma (SRM 1950) were also prepared as external quality control samples. Briefly, 400 µL of methanol containing 500 ng/mL L-tryptophan-d5 was vortexed at 5,000 rpm for 2 min. Afterwards, extracts were centrifuged at 16,000 rcf for 10 min at 4 °C, and 350 µL of the supernatant was dried by vacuum concentrator overnight and reconstituted with 100 µL water-methanol (95:5, v/v) prior to analysis. Study samples were randomized before sample preparation and data acquisition. Quality control study pools, NIST plasma references, and blanks were interspersed at a rate of 10% amongst the study samples during the analysis sequence. Samples were run across three batches.

The untargeted data was acquired on a Vanquish UHPLC system coupled with a Q Exactive™ HF-X Hybrid Quadrupole-Orbitrap Mass Spectrometer (Thermo Fisher Scientific, San Jose, CA). A volume of 5 µl was injected for analysis. Metabolites were separated via an HSS T3 C18 column (2.1 × 100 mm, 1.7 µm, Waters Corporation) at 50 °C with mobile phases of water (A) and methanol (B), each containing 0.1% formic acid (v/v). The UHPLC linear gradient began with 2% B, and increased to 100% B in 16 min, then held for 4 min, with a flow rate at 400 µL/min. The untargeted metabolomics data was acquired in a range of 70-1050 m/z using the data dependent acquisition mode for the MS/MS spectra data.

The UHPLC-HR-MS data were processed by Progenesis QI (version 2.1, Waters Corporation) for peak identification and alignment. Background signals were excluded if the mean intensity across blanks were higher than that across the quality control study pools based on the unnormalized data^120^. The remaining peaks were normalized by Progenesis QI using the “normalize to all” feature. Signals that significantly differed amongst the three running batches (ANOVA, with false discovery rate (FDR) correction *q* < 0.05) were excluded for further analysis. Finally, signals were filtered to only include those detected in 95% of serum samples and the metabolomics data was log_2_-transformed to normalize positive skewness.

### Statistical analysis

All statistical analyses were conducted in R (Boston, MA, USA, Version 4.1.0). Serum PFAS concentrations were first analyzed with descriptive statistics, including their detection frequency, distribution percentile, and geometric mean (GM) and standard deviation (GSD). For serum samples with nondetectable PFAS concentrations (<5%), the exposure values were imputed with the limit of detection (LOD) divided by $\sqrt{2}$^121^. The serum PFAS concentrations were positively skewed and log_2_-transformed to normalize their distribution. For every pair of log_2_-transformed PFAS in this study, Pearson correlations were examined. Descriptive statistics were also performed for the clinical, sociodemographic, and anthropometric variables. Continuous variables are presented as the mean ± standard deviation (SD) and categorical variables are presented as the count and relative frequency.

We tested for collinearity among variables by examining bivariate associations. Other assumptions to proceed with regression models were satisfied by visually inspecting distributions of the variables. Multivariable regression models were used to investigate individual maternal PFAS concentrations in relation to individual birth outcomes. All of the models were adjusted for the same set of covariates: maternal age at enrollment, maternal prenatal BMI, maternal prenatal tobacco and marijuana use, parity, maternal level of education, neonatal sex, and gestational week at sample collection. Linear regressions were fitted to the continuous birth outcome, gestational weeks at birth, while logistic regressions were fitted to the binary birth outcomes (PTB, ETB, spontaneous or medically indicated delivery). For logistic regression models, the reference group was healthy, full-term newborns (i.e., born to mothers without a diagnosis of preeclampsia, gestational diabetes, or gestational hypertension (N = 118)). We also modeled dose-response relationships between quartiles of serum PFAS concentrations and birth outcomes.

Nine MWAS were carried out to determine the newborn DBS signals associated with the prenatal PFAS concentrations and birth outcomes. First, the exposure-metabolomic signal relationships were investigated for each PFAS species by fitting a linear model with the following form:

$${\log }_{2}\left({{\mbox{Intensity}}}\right)=	 {{{{\rm{\beta }}}}}_{0}+{{{{\rm{\beta }}}}}_{1}{\log }_{2}\left({{\mbox{PFAS}}}\right)+{{{{\rm{\beta }}}}}_{2}{{\mbox{Maternal}}} \, {age}+{{{{\rm{\beta }}}}}_{3}{{\mbox{Prenatal}}} \, {BMI} \\ 	+{{{{\rm{\beta }}}}}_{4}{{\mbox{Parity}}}+{{{{\rm{\beta }}}}}_{5}{{\mbox{Education}}}{+{{{\rm{\beta }}}}}_{6}{{\mbox{Tobacco}}} \, {use} \\ 	+{{{{\rm{\beta }}}}}_{7}{{\mbox{Marijuana}}} \, {use}+{{{{\rm{\beta }}}}}_{8}{{\mbox{Neonatal}}} \, {sex} \\ 	+{{{{\rm{\beta }}}}}_{9}{{\mbox{Gestational}}} \,{week} \, {PFAS} \,{sample} \, {collected}+\in $$

where *Intensity* is the signal for every metabolomic signal detected in the DBS after data filtering, ${\beta }_{0}$ is the intercept, ${\beta }_{1}$ is the PFAS regression estimate coefficient, and ${\beta }_{2-9}$ are the regression estimate coefficients for the covariates. A similar modeling approach was applied to investigate the metabolomic signal-outcome relationships for continuous gestational age:

$${{\mbox{Gestational}}} \,{age}=	 {{{{\rm{\beta }}}}}_{0}+{{{{\rm{\beta }}}}}_{1}{\log }_{2}\left({{\mbox{Intensity}}}\right)+{{{{\rm{\beta }}}}}_{2}{{\mbox{Maternal}}} \,{age} \\ 	+{{{{\rm{\beta }}}}}_{3}{{\mbox{Prenatal}}} \, {BMI}+{{{{\rm{\beta }}}}}_{4}{{\mbox{Parity}}}+{{{{\rm{\beta }}}}}_{5}{{\mbox{Education}}}\\ 	 {+{{{\rm{\beta }}}}}_{6}{{\mbox{Tobacco}}} \,{use}+{{{{\rm{\beta }}}}}_{7}{{\mbox{Marijuana}}} \, {use}+{{{{\rm{\beta }}}}}_{8}{{\mbox{Neonatal}}} \, {sex}+\in $$

where ${\beta }_{1}$ is now the regression estimate coefficient for the *Intensity* of every metabolomic signal. The same set of covariates was included in the models fitted for binary birth outcomes as shown below:

$${{\mathrm{ln}}}\left(\frac{{{\mbox{P}}}({{\mbox{Birth}}} \,{outcome})}{1-{{\mbox{P}}}({{\mbox{Birth}}} \,{outcome})}\right)=	 {{{{\rm{\beta }}}}}_{0}+{{{{\rm{\beta }}}}}_{1}{\log }_{2}\left({{\mbox{Intensity}}}\right)+{{{{\rm{\beta }}}}}_{2}{{\mbox{Maternal}}} \,{age} \\ 	+{{{{\rm{\beta }}}}}_{3}{{\mbox{Prenatal}}} \,{BMI}+{{{{\rm{\beta }}}}}_{4}{{\mbox{Parity}}}+{{{{\rm{\beta }}}}}_{5}{{\mbox{Education}}}\\ 	{+{{{\rm{\beta }}}}}_{6}{{\mbox{Tobacco}}} \,{use}+{{{{\rm{\beta }}}}}_{7}{{\mbox{Marijuana}}} \,{use} \\ 	+{{{{\rm{\beta }}}}}_{8}{{\mbox{Neonatal}}} \,{sex}$$

The *p*-values obtained from the MWAS were corrected for FDR using the Benjamini-Hochberg and Bonferroni procedures since multiple comparisons were made^122,123^.

### Pathway enrichment analysis

Pathway enrichment analyses were performed using the bioinformatics software *Mummichog* (Version 1.0.10), which predicts biological networks, pathways, and metabolites based on significant signals with tentative chemical identities^124^. We serially reduced the significance level at which signals were eligible for inclusion in the pathway enrichment analyses, ranging from a raw *p*-value < 0.05 to Bonferroni corrected *q*-value < 0.01 as long as there were ≥100 signals available. Additionally, to minimize the chance of false positive discoveries, we only retained pathways with *Mummichog*’s $\gamma$-adjusted *p*-value < 0.05 after 1000 permutations were performed and pathways enriched with ≥10% overlapping signals relative to the pathway size.

### Chemical identification and annotation

Signals were identified by matching against the NC HHEAR Hub in-house experimental standards library (IESL) generated from acquired data for over 2400 compounds, including endogenous metabolites associated with host metabolism and exogenous metabolites related to lifetime exposures including environmentally relevant compounds, ingested food components, drugs and medications, as well as derivatives/conjugates formed in vivo after exposure. All standard reference compounds in the IESL were run under identical conditions to study samples. The same set of signals were also matched to public databases, including NIST and METLIN. These signals were matched with metabolites and labeled with ontology levels (OL) to indicate the evidence that supported the identification or annotation, including RT, exact mass (MS), MS/MS fragmentation pattern, and/or isotopic ion pattern^125–127^. Metabolites that matched to the IESL by RT (± 0.5 min), MS ( <5 ppm), and MS/MS (similarity score >30) are labeled OL1, whereas those that match by RT and MS are labeled OL2a. Metabolites with OL1 and OL2a were identified with the highest level of confidence in this study. An OL2b label was provided for signals matched by MS and MS/MS to the IESL but were outside the RT window (±0.5 min). Metabolites annotated with OL2b are the conjugates/isomers that share similar or the same moieties with the matched compound in IESL. A public database (PDa) label was provided for signals matched by MS (<5 ppm) and experimental MS/MS (similarity score >30) to public databases. Annotated signals with a PDa label could be the listed compound, isomer, or derivative of the listed compound. OL2b and PDa were considered confident annotations in this study. Metabolites annotated with less confidence were not retained in subsequent analyses.

### Meet-in-the-middle analysis

We analyzed the neonatal metabolome for molecular signatures underlying the relationship between maternal PFAS concentrations and gestational age at birth outcomes with the MITM framework^39^. Specifically, the pathways and metabolites significantly enriched in both the PFAS MWAS and birth outcomes MWAS were identified as intermediate biological mechanisms and biomarkers. These results informed our construction of a hypothesized diagram relating the effect of prenatal PFAS exposure on birth outcomes in our study population.

### Sensitivity analysis

The PFAS body burden in fetal tissues has been shown to increase with gestational age and is believed to coincide with trans-placental transfer^29,128,129^. To assess the influence of this hypothesized confounder on the enriched signals and pathways associated with PFAS exposure, we conducted sensitivity analyses without adjusting for the week when the maternal serum sample was collected and compared the results to those obtained in the main analyses. We also performed sensitivity analyses to identify similarities across the enriched pathways associated with PFAS and birth outcomes at the serially reduced significance thresholds, as described above.

### NIH funding disclaimer

The content is solely the responsibility of the authors and does not necessarily represent the official views of the National Institutes of Health.

### Reporting summary

Further information on research design is available in the Nature Portfolio Reporting Summary linked to this article.

## Supplementary information

Supplementary Information File

Reporting Summary

## Data Availability

The raw and processed metabolomics data generated in this study have been deposited in the Metabolomics Workbench (https://www.metabolomicsworkbench.org/ Study ID ST002692) by the UNC Human Health Exposure Analysis Resource (HHEAR) Laboratory. The clinical outcome and PFAS exposure data are available under restricted access to protect the privacy of the study participants, access can be obtained by emailing corresponding authors Drs. Liang and Dunlop. Requests will be responded within 10 business days. The demographic covariates data are protected and are not available due to data privacy laws. All the source data for figures and tables, coding materials, and data protocols are provided in the Supplementary Information/Source Data file. Source data are provided with this paper.

## Source data

Source Data

## Code availability

Code is available from the corresponding author and request will be responded within 10 business days.

## References

[1] Osterman M, Hamilton B, Martin JA, Driscoll AK, Valenzuela CP (2021). Births: final data for 2020. Natl Vital Stat Rep..

[2] Crump C, Sundquist K, Winkleby MA, Sundquist J (2013). Early-term birth (37-38 weeks) and mortality in young adulthood. Epidemiology..

[3] Moster D, Lie RT, Markestad T (2008). Long-term medical and social consequences of preterm birth. N. Engl. J. Med..

[4] Group E (2009). One-year survival of extremely preterm infants after active perinatal care in Sweden. JAMA..

[5] McCormick MC, Litt JS, Smith VC, Zupancic JA (2011). Prematurity: an overview and public health implications. Annu. Rev. Public Health.

[6] Gillman MW (2005). Developmental origins of health and disease. N. Engl. J. Med..

[7] Barber LE, Bertrand KA, Rosenberg L, Battaglia TA, Palmer JR (2019). Pre- and perinatal factors and incidence of breast cancer in the Black Women’s Health Study. Cancer Causes Control..

[8] Li S (2014). Preterm birth and risk of type 1 and type 2 diabetes: systematic review and meta-analysis. Obes. Rev..

[9] McCarton CM, Vaughan HG (1984). Perinatal variables and neurodevelopmental outcomes with preterm births. Clin. Obstet. Gynecol..

[10] Wadhwa PD, Buss C, Entringer S, Swanson JM (2009). Developmental origins of health and disease: brief history of the approach and current focus on epigenetic mechanisms. Semin. Reprod. Med..

[11] Barker DJ (1998). In utero programming of chronic disease. Clin. Sci. (Lond.).

[12] Svensson AC (2009). Maternal effects for preterm birth: a genetic epidemiologic study of 630,000 families. Am. J. Epidemiol..

[13] D’Onofrio BM (2013). Preterm birth and mortality and morbidity: a population-based quasi-experimental study. JAMA Psychiatry.

[14] Clausson B, Lichtenstein P, Cnattingius S (2000). Genetic influence on birthweight and gestational length determined by studies in offspring of twins. BJOG..

[15] Wilcox AJ, Skjaerven R, Lie RT (2008). Familial patterns of preterm delivery: maternal and fetal contributions. Am. J. Epidemiol..

[16] Institute of Medicine (US) Committee on Understanding Premature Birth and Assuring Healthy Outcomes. in *Preterm Birth: Causes, Consequences, and Prevention* (eds Behrman, R. E. & Butler, A. S.) *Thee National Academies Collection: Reports funded by National Institutes of Health*. (Institute of Medicine (US) Committee on Understanding Premature Birth and Assuring Healthy Outcomes, 2007)

[17] Blake BE, Fenton SE (2020). Early life exposure to per- and polyfluoroalkyl substances (PFAS) and latent health outcomes: a review including the placenta as a target tissue and possible driver of peri- and postnatal effects. Toxicology.

[18] Liew Z, Goudarzi H, Oulhote Y (2018). Developmental exposures to perfluoroalkyl substances (PFASs): an update of associated health outcomes. Curr. Environ. Health Rep..

[19] Lau C, Butenhoff JL, Rogers JM (2004). The developmental toxicity of perfluoroalkyl acids and their derivatives. Toxicol. Appl. Pharmacol..

[20] Olsen GW, Butenhoff JL, Zobel LR (2009). Perfluoroalkyl chemicals and human fetal development: an epidemiologic review with clinical and toxicological perspectives. Reprod. Toxicol..

[21] Moody CA, Field JA (1999). Determination of perfluorocarboxylates in groundwater impacted by fire-fighting activity. Environ. Sci. Technol..

[22] Han X, Snow TA, Kemper RA, Jepson GW (2003). Binding of perfluorooctanoic acid to rat and human plasma proteins. Chem. Res. Toxicol..

[23] Forsthuber M (2020). Albumin is the major carrier protein for PFOS, PFOA, PFHxS, PFNA and PFDA in human plasma. Environ. Int..

[24] Khazaee M (2021). Perfluoroalkyl acid binding with peroxisome proliferator-activated receptors alpha, gamma, and delta, and fatty acid binding proteins by equilibrium dialysis with a comparison of methods. Toxics.

[25] Sunderland EM (2019). A review of the pathways of human exposure to poly- and perfluoroalkyl substances (PFASs) and present understanding of health effects. J. Expo Sci. Environ. Epidemiol..

[26] Woodruff TJ, Zota AR, Schwartz JM (2011). Environmental chemicals in pregnant women in the United States: NHANES 2003-2004. Environ. Health Perspect..

[27] Chang CJ (2021). Serum per- and polyfluoroalkyl substance (PFAS) concentrations and predictors of exposure among pregnant African American women in the Atlanta area, Georgia. Environ. Res..

[28] Chen F, Yin S, Kelly BC, Liu W (2017). Chlorinated polyfluoroalkyl ether sulfonic acids in matched maternal, cord, and placenta samples: a study of transplacental transfer. Environ. Sci. Technol..

[29] Chen F, Yin S, Kelly BC, Liu W (2017). Isomer-specific transplacental transfer of perfluoroalkyl acids: results from a survey of paired maternal, cord sera, and placentas. Environ. Sci. Technol..

[30] Wang Y (2019). Efficiency of maternal-fetal transfer of perfluoroalkyl and polyfluoroalkyl substances. Environ. Sci. Pollut. Res. Int..

[31] Yang L (2016). Human placental transfer of perfluoroalkyl acid precursors: Levels and profiles in paired maternal and cord serum. Chemosphere.

[32] Huo X (2020). Perfluoroalkyl substances exposure in early pregnancy and preterm birth in singleton pregnancies: a prospective cohort study. Environ. Health.

[33] Manzano-Salgado CB (2017). Prenatal exposure to perfluoroalkyl substances and birth outcomes in a Spanish birth cohort. Environ. Int..

[34] Meng Q, Inoue K, Ritz B, Olsen J, Liew Z (2018). Prenatal exposure to perfluoroalkyl substances and birth outcomes; an updated analysis from the danish national birth cohort. Int. J. Environ. Res. Public Health.

[35] Casals-Casas C, Desvergne B (2011). Endocrine disruptors: from endocrine to metabolic disruption. Annu. Rev. Physiol..

[36] Heindel JJ (2017). Metabolism disrupting chemicals and metabolic disorders. Reprod. Toxicol..

[37] Chang CJ (2021). Per- and polyfluoroalkyl substance (PFAS) exposure, maternal metabolomic perturbation, and fetal growth in African American women: a meet-in-the-middle approach. Environ. Int..

[38] Starling AP (2017). Perfluoroalkyl substances during pregnancy and offspring weight and adiposity at birth: examining mediation by maternal fasting glucose in the healthy start study. Environ. Health Perspect..

[39] Chadeau-Hyam M (2011). Meeting-in-the-middle using metabolic profiling—a strategy for the identification of intermediate biomarkers in cohort studies. Biomarkers.

[40] Lankadurai, B., G. Nagato, E. G. & Simpson, M. J. Environmental metabolomics: an emerging approach to study organism responses to environmental stressors. *Environ. Rev.* **21**, 180–205 (2013).

[41] Gaskins AJ (2021). Periconception air pollution, metabolomic biomarkers, and fertility among women undergoing assisted reproduction. Environ. Int..

[42] Tan Y (2022). High-resolution metabolomics of exposure to tobacco smoke during pregnancy and adverse birth outcomes in the Atlanta African American maternal-child cohort. Environ. Pollut..

[43] Gui SY (2022). Association between exposure to per- and polyfluoroalkyl substances and birth outcomes: a systematic review and meta-analysis. Front. Public Health.

[44] Eick SM (2022). Prenatal PFAS and psychosocial stress exposures in relation to fetal growth in two pregnancy cohorts: Applying environmental mixture methods to chemical and non-chemical stressors. Environ. Int..

[45] Preston EV (2022). Early-pregnancy plasma per- and polyfluoroalkyl substance (PFAS) concentrations and hypertensive disorders of pregnancy in the Project Viva cohort. Environ. Int..

[46] Bommarito PA, Ferguson KK, Meeker JD, McElrath TF, Cantonwine DE (2021). Maternal levels of perfluoroalkyl substances (PFAS) during early pregnancy in relation to preeclampsia subtypes and biomarkers of preeclampsia risk. Environ Health Perspect..

[47] Starling AP (2019). Prenatal exposure to per- and polyfluoroalkyl substances and infant growth and adiposity: the Healthy Start Study. Environ. Int..

[48] Gao X (2021). Per- and polyfluoroalkyl substances exposure during pregnancy and adverse pregnancy and birth outcomes: a systematic review and meta-analysis. Environ. Res..

[49] Sagiv SK (2018). Early-pregnancy plasma concentrations of perfluoroalkyl substances and birth outcomes in project viva: confounded by pregnancy hemodynamics?. Am. J. Epidemiol..

[50] Chen MH (2012). Perfluorinated compounds in umbilical cord blood and adverse birth outcomes. PLoS ONE.

[51] Gardener H, Sun Q, Grandjean P (2021). PFAS concentration during pregnancy in relation to cardiometabolic health and birth outcomes. Environ. Res..

[52] Liu X (2020). Does low maternal exposure to per- and polyfluoroalkyl substances elevate the risk of spontaneous preterm birth? a nested case-control study in China. Environ. Sci. Technol..

[53] Eick SM (2020). Associations between prenatal maternal exposure to per- and polyfluoroalkyl substances (PFAS) and polybrominated diphenyl ethers (PBDEs) and birth outcomes among pregnant women in San Francisco. Environ Health.

[54] Lee YJ, Jung HW, Kim HY, Choi YJ, Lee YA (2021). Early-life exposure to per- and poly-fluorinated alkyl substances and growth, adiposity, and puberty in children: a systematic review. Front. Endocrinol. (Lausanne).

[55] Robinson JF, Hamilton EG, Lam J, Chen H, Woodruff TJ (2020). Differences in cytochrome p450 enzyme expression and activity in fetal and adult tissues. Placenta.

[56] Hines RN (2007). Ontogeny of human hepatic cytochromes P450. J. Biochem. Mol. Toxicol..

[57] Omura T (2006). Mitochondrial P450s. Chem. Biol. Interact..

[58] Hu X, Go YM, Jones DP (2020). Omics integration for mitochondria systems biology. Antioxid. Redox Signal..

[59] Chang CJ (2021). Associations of single and multiple per- and polyfluoroalkyl substance (PFAS) exposure with vitamin D biomarkers in African American women during pregnancy. Environ. Res..

[60] Sinisalu L (2021). Prenatal exposure to poly-/per-fluoroalkyl substances is associated with alteration of lipid profiles in cord-blood. Metabolomics..

[61] Roth K (2021). Exposure to a mixture of legacy, alternative, and replacement per- and polyfluoroalkyl substances (PFAS) results in sex-dependent modulation of cholesterol metabolism and liver injury. Environ. Int..

[62] Salihovic S (2020). Simultaneous determination of perfluoroalkyl substances and bile acids in human serum using ultra-high-performance liquid chromatography-tandem mass spectrometry. Anal. Bioanal. Chem..

[63] Etzel TM, Braun JM, Buckley JP (2019). Associations of serum perfluoroalkyl substance and vitamin D biomarker concentrations in NHANES, 2003-2010. Int. J. Hyg. Environ. Health.

[64] Ding N, Harlow SD, Randolph JF, Loch-Caruso R, Park SK (2020). Perfluoroalkyl and polyfluoroalkyl substances (PFAS) and their effects on the ovary. Hum. Reprod. Update.

[65] Di Nisio A (2019). Endocrine disruption of androgenic activity by perfluoroalkyl substances: clinical and experimental evidence. J. Clin. Endocrinol. Metab..

[66] Du G (2013). Endocrine-related effects of perfluorooctanoic acid (PFOA) in zebrafish, H295R steroidogenesis and receptor reporter gene assays. Chemosphere.

[67] Wang Y (2021). Perfluoroalkyl substances and sex hormones in postmenopausal women: NHANES 2013-2016. Environ. Int..

[68] Makarov MV, Trammell SAJ, Migaud ME (2019). The chemistry of the vitamin B3 metabolome. Biochem. Soc. Trans..

[69] Yang Y, Sauve AA (2016). NAD(+) metabolism: bioenergetics, signaling and manipulation for therapy. Biochim. Biophys. Acta.

[70] Kim HK, Han J (2020). Tetrahydrobiopterin in energy metabolism and metabolic diseases. Pharmacol. Res..

[71] Kwon NS (1990). L-citrulline production from L-arginine by macrophage nitric oxide synthase. The ureido oxygen derives from dioxygen. J. Biol. Chem..

[72] Daubner SC, Le T, Wang S (2011). Tyrosine hydroxylase and regulation of dopamine synthesis. Arch. Biochem. Biophys..

[73] Thony B, Auerbach G, Blau N (2000). Tetrahydrobiopterin biosynthesis, regeneration and functions. Biochem. J..

[74] Bendall JK, Douglas G, McNeill E, Channon KM, Crabtree MJ (2014). Tetrahydrobiopterin in cardiovascular health and disease. Antioxid. Redox Signal.

[75] Wielsoe M, Long M, Ghisari M, Bonefeld-Jorgensen EC (2015). Perfluoroalkylated substances (PFAS) affect oxidative stress biomarkers in vitro. Chemosphere..

[76] Ojo AF, Xia Q, Peng C, Ng JC (2021). Evaluation of the individual and combined toxicity of perfluoroalkyl substances to human liver cells using biomarkers of oxidative stress. Chemosphere.

[77] Sonkar R, Kay MK, Choudhury M (2019). PFOS modulates interactive epigenetic regulation in first-trimester human trophoblast cell line HTR-8/SVneo. Chem. Res.Toxicol..

[78] Li D (2019). Maternal exposure to perfluorooctanoic acid (PFOA) causes liver toxicity through PPAR-alpha pathway and lowered histone acetylation in female offspring mice. Environ. Sci. Pollut. Res. Int..

[79] Aung MT (2019). Prediction and associations of preterm birth and its subtypes with eicosanoid enzymatic pathways and inflammatory markers. Sci. Rep..

[80] Regnault TR, de Vrijer B, Battaglia FC (2002). Transport and metabolism of amino acids in placenta. Endocrine.

[81] Manta-Vogli PD, Schulpis KH, Dotsikas Y, Loukas YL (2020). The significant role of amino acids during pregnancy: nutritional support. J. Matern. Fetal Neonatal. Med..

[82] Wan HT, Wong AY, Feng S, Wong CK (2020). Effects of in utero exposure to perfluorooctane sulfonate on placental functions. Environ. Sci. Technol..

[83] Silvano A (2021). Tryptophan metabolism and immune regulation in the human placenta. J. Reprod. Immunol..

[84] Badawy AA (2015). Tryptophan metabolism, disposition and utilization in pregnancy. Biosci. Rep..

[85] Rosenfeld CS (2021). The placenta-brain-axis. J. Neurosci. Res..

[86] Goeden N (2016). Maternal inflammation disrupts fetal neurodevelopment via increased placental output of serotonin to the fetal brain. J. Neurosci..

[87] Money KM, Stanwood GD (2013). Developmental origins of brain disorders: roles for dopamine. Front. Cell Neurosci..

[88] Sinisalu L (2020). Early-life exposure to perfluorinated alkyl substances modulates lipid metabolism in progression to celiac disease. Environ. Res..

[89] Lin CY, Lee HL, Hwang YT, Su TC (2020). The association between total serum isomers of per- and polyfluoroalkyl substances, lipid profiles, and the DNA oxidative/nitrative stress biomarkers in middle-aged Taiwanese adults. Environ. Res..

[90] Blomberg AJ (2021). Early-life associations between per- and polyfluoroalkyl substances and serum lipids in a longitudinal birth cohort. Environ. Res..

[91] Canova C (2020). Associations between perfluoroalkyl substances and lipid profile in a highly exposed young adult population in the Veneto Region. Environ. Int..

[92] Frisbee SJ (2010). Perfluorooctanoic acid, perfluorooctanesulfonate, and serum lipids in children and adolescents: results from the C8 Health Project. Arch. Pediatr. Adolesc. Med..

[93] Wan HT (2012). PFOS-induced hepatic steatosis, the mechanistic actions on beta-oxidation and lipid transport. Biochim. Biophys. Acta..

[94] Szilagyi JT, Avula V, Fry RC (2020). Perfluoroalkyl substances (PFAS) and their effects on the placenta, pregnancy, and child development: a potential mechanistic role for placental peroxisome proliferator-activated receptors (PPARs). Curr. Environ. Health Rep..

[95] Yu N (2016). Effects of perfluorooctanoic acid on metabolic profiles in brain and liver of mouse revealed by a high-throughput targeted metabolomics approach. Sci. Rep..

[96] Wang J, Yan S, Zhang W, Zhang H, Dai J (2015). Integrated proteomic and miRNA transcriptional analysis reveals the hepatotoxicity mechanism of PFNA exposure in mice. J. Proteome Res..

[97] Tan F (2012). Global liver proteome analysis using iTRAQ labeling quantitative proteomic technology to reveal biomarkers in mice exposed to perfluorooctane sulfonate (PFOS). Environ. Sci. Technol..

[98] Ding L (2009). Systems biological responses to chronic perfluorododecanoic acid exposure by integrated metabonomic and transcriptomic studies. J. Proteome Res..

[99] Peiris HN, Vaswani K, Almughlliq F, Koh YQ, Mitchell MD (2017). Review: eicosanoids in preterm labor and delivery: potential roles of exosomes in eicosanoid functions. Placenta.

[100] ACOG Committee Opinion No 579: Definition of term pregnancy. *Obstet Gynecol.* **122**, 1139–1140 (2013). 10.1097/01.AOG.0000437385.88715.4a 24150030

[101] Marks KJ (2019). Maternal serum concentrations of perfluoroalkyl substances during pregnancy and gestational weight gain: The Avon Longitudinal Study of Parents and Children. Reprod. Toxicol..

[102] Romano ME (2021). Per- and polyfluoroalkyl substance mixtures and gestational weight gain among mothers in the Health Outcomes and Measures of the Environment study. Int. J. Hyg. Environ. Health.

[103] Lehmann S, Delaby C, Vialaret J, Ducos J, Hirtz C (2013). Current and future use of “dried blood spot” analyses in clinical chemistry. Clin. Chem. Lab Med..

[104] Velghe S, Delahaye L, Stove CP (2019). Is the hematocrit still an issue in quantitative dried blood spot analysis?. J. Pharm. Biomed. Anal..

[105] Vespasiani-Gentilucci U (2017). Platelet count may impact on lysosomal acid lipase activity determination in dried blood spot. Clin. Biochem..

[106] Jain A (2022). Hemoglobin normalization outperforms other methods for standardizing dried blood spot metabolomics: a comparative study. Sci. Total Environ..

[107] Corwin EJ (2017). Protocol for the emory University African American Vaginal, Oral, and Gut Microbiome in Pregnancy Cohort Study. BMC Pregnancy Childbirth.

[108] Brennan PA (2019). Protocol for the Emory University African American maternal stress and infant gut microbiome cohort study. BMC Pediatr..

[109] Honda M, Robinson M, Kannan K (2018). A rapid method for the analysis of perfluorinated alkyl substances in serum by hybrid solid-phase extraction. Environ. Chem..

[110] Balshaw DM, Collman GW, Gray KA, Thompson CL (2017). The Children’s Health Exposure Analysis Resource: enabling research into the environmental influences on children’s health outcomes. Curr. Opin. Pediatr..

[111] Viet SM (2021). Human Health Exposure Analysis Resource (HHEAR): a model for incorporating the exposome into health studies. Int. J. Hyg. Environ. Health.

[112] Kannan K (2021). Quality assurance and harmonization for targeted biomonitoring measurements of environmental organic chemicals across the Children’s Health Exposure Analysis Resource laboratory network. Int. J. Hyg. Environ. Health.

[113] Committee opinion no 611. Method for estimating due date. *Obstet Gynecol.* **124**, 863–866 (2014). 10.1097/01.AOG.0000454932.15177.be 25244460

[114] Committee opinion no 700. Methods for estimating the due date. *Obstet Gynecol.* **129**, e150–e154 (2017). 10.1097/AOG.0000000000002046 28426621

[115] Gong ZG, Hu J, Wu X, Xu YJ (2017). The recent developments in sample preparation for mass spectrometry-based metabolomics. Crit. Rev. Anal. Chem..

[116] Petrick L (2017). An untargeted metabolomics method for archived newborn dried blood spots in epidemiologic studies. Metabolomics.

[117] Zhuang YJ, Mangwiro Y, Wake M, Saffery R, Greaves RF (2022). Multi-omics analysis from archival neonatal dried blood spots: limitations and opportunities. Clin. Chem. Lab Med..

[118] Ward C (2021). Nontargeted mass spectrometry of dried blood spots for interrogation of the human circulating metabolome. J. Mass Spectrom..

[119] GDoPH. NBS Policies and Procedures. https://dph.georgia.gov/NBS/nbs-policies-and-procedures (2023)

[120] Li S (2021). Multi-omics analysis of glucose-mediated signaling by a moonlighting Gbeta protein Asc1/RACK1. PLoS Genet..

[121] Hornung RW, Reed LD (1990). Estimation of average concentration in the presence of nondetectable values. Appl. Occupational Environ. Hyg..

[122] Benjamini Y, Hochberg Y (1995). Controlling the false discovery rate: a practical and powerful approach to multiple testing. J. Roy. Statistical Soc: Ser. B (Methodological)..

[123] Bland JM, Altman DG (1995). Multiple significance tests: the Bonferroni method. BMJ..

[124] Li S (2013). Predicting network activity from high throughput metabolomics. PLoS Comput. Biol..

[125] Harville EW (2021). Untargeted analysis of first trimester serum to reveal biomarkers of pregnancy complications: a case-control discovery phase study. Sci. Rep..

[126] Li YY (2020). Untargeted metabolomics: biochemical perturbations in golestan cohort study opium users inform intervention strategies. Front. Nutr..

[127] Ghanbari R (2021). Metabolomics reveals biomarkers of opioid use disorder. Transl. Psychiatry..

[128] Mamsen LS (2019). Concentrations of perfluoroalkyl substances (PFASs) in human embryonic and fetal organs from first, second, and third trimester pregnancies. Environ. Int..

[129] Cai D (2020). High trans-placental transfer of perfluoroalkyl substances alternatives in the matched maternal-cord blood serum: evidence from a birth cohort study. Sci. Total Environ..

