# Supplementary material for: Newborn metabolomic signatures of maternal per- and polyfluoroalkyl substance exposure and reduced length of gestation
Source: Nat Commun. 2023 May 30;14:3120. doi: 10.1038/s41467-023-38710-3 (PMC10229585; doi:10.1038/s41467-023-38710-3)
Supplement: Supplementary file 1 — Supplementary Information File [file 41467_2023_38710_MOESM1_ESM.pdf]

Supplemental Material

Newborn Metabolomic Signatures of Maternal Serum Per- and Polyfluoroalkyl Substance Levels and Reduced Length of Gestation: A Prospective Analysis in the Atlanta African American Maternal-Child Cohort

Figure S1. Flowchart of 267 participants included in this study.

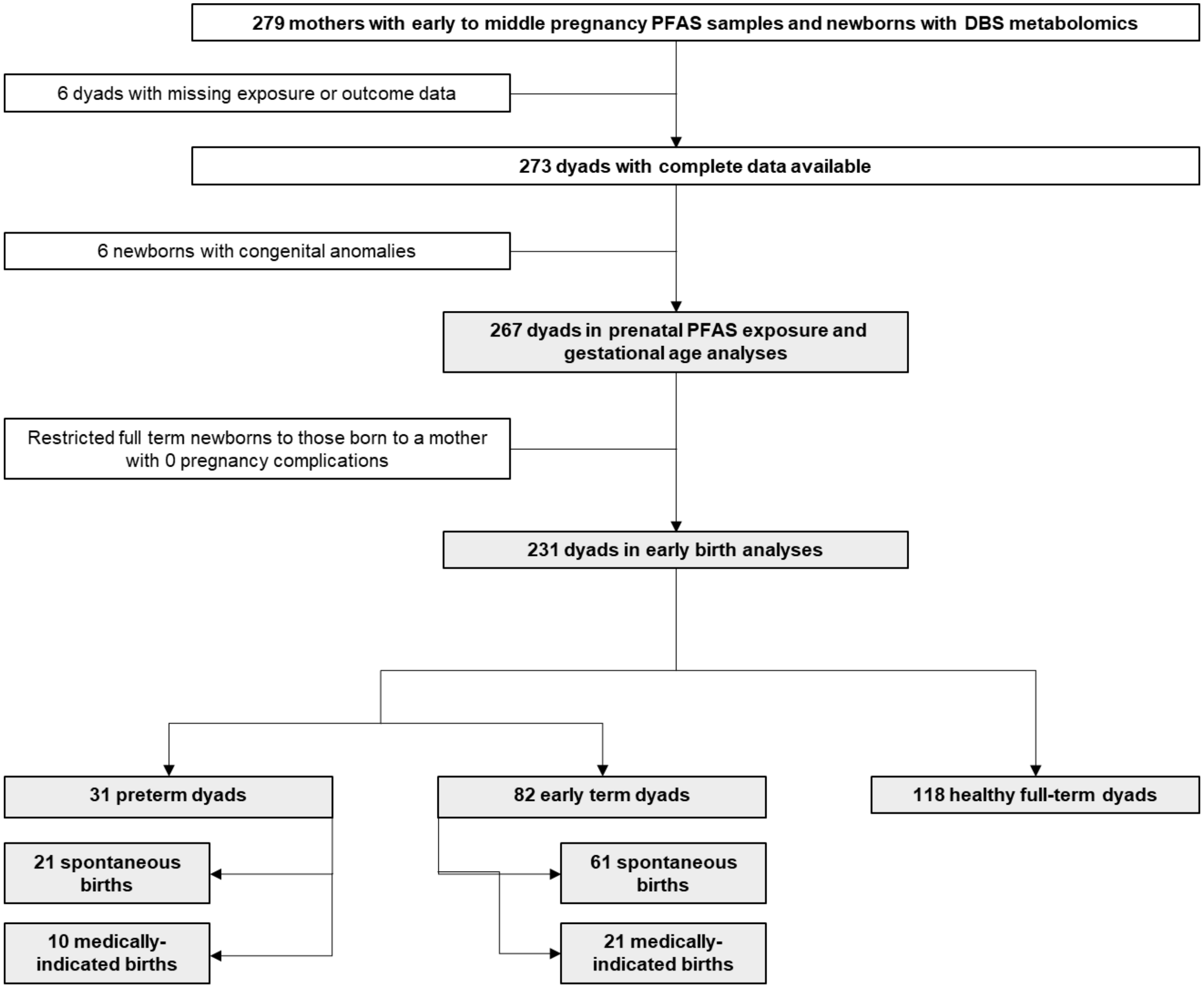

Abbreviations: PFAS = perfluoroalkyl substances; DBS = dried blood spot.

Figure S2: Directed acyclic graph (DAG) showing the hypothesized relationships between prenatal maternal PFAS exposure, gestational age at birth outcomes, newborn dried blood spot metabolome, and potential confounders in this study.

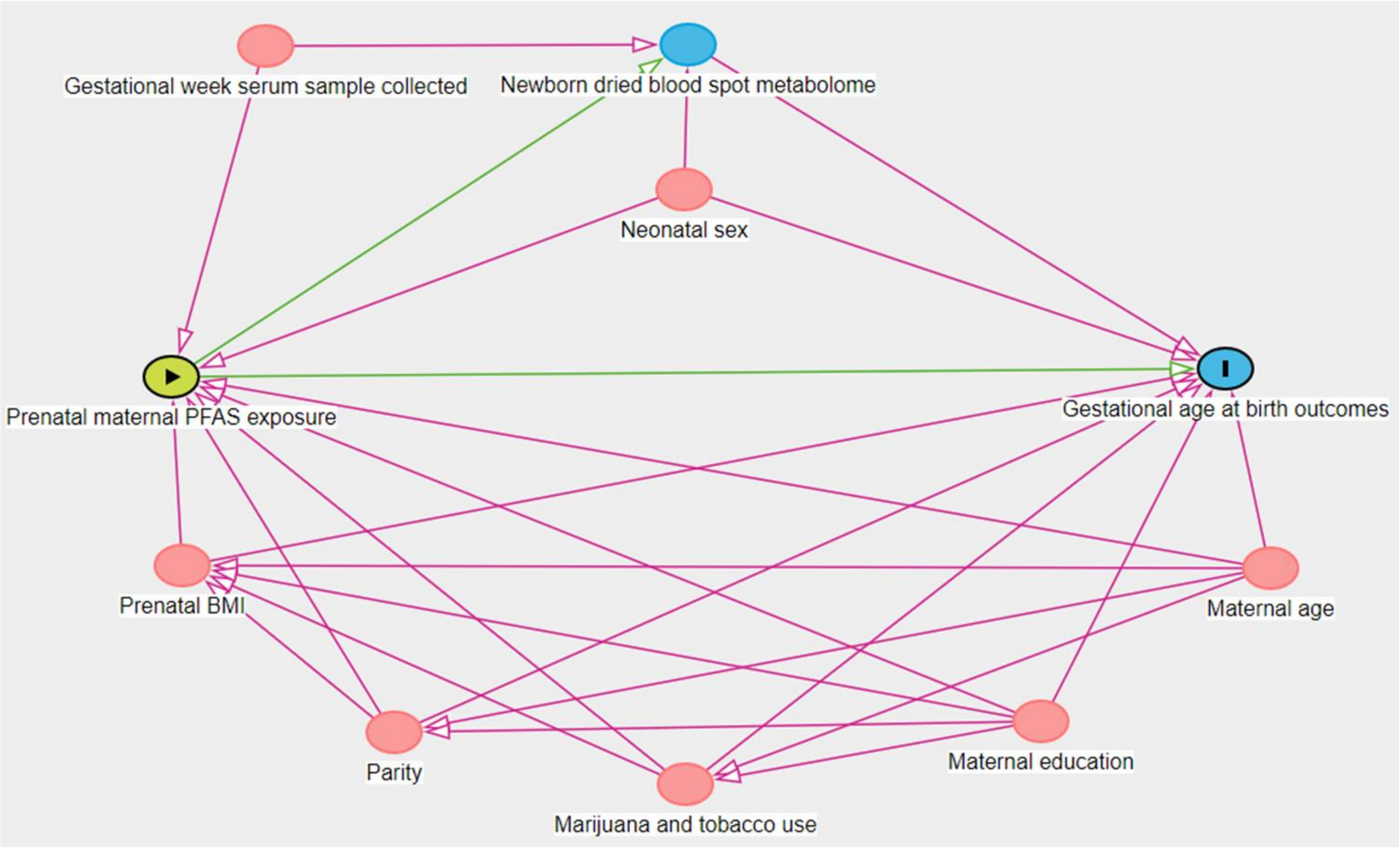

Figure S3. Analytical flow of *meet-in-the-middle* analysis to identify pathways and metabolites underlying the relationship between prenatal PFAS exposure and gestational age at birth outcomes in Atlanta, 2016 – 2020.

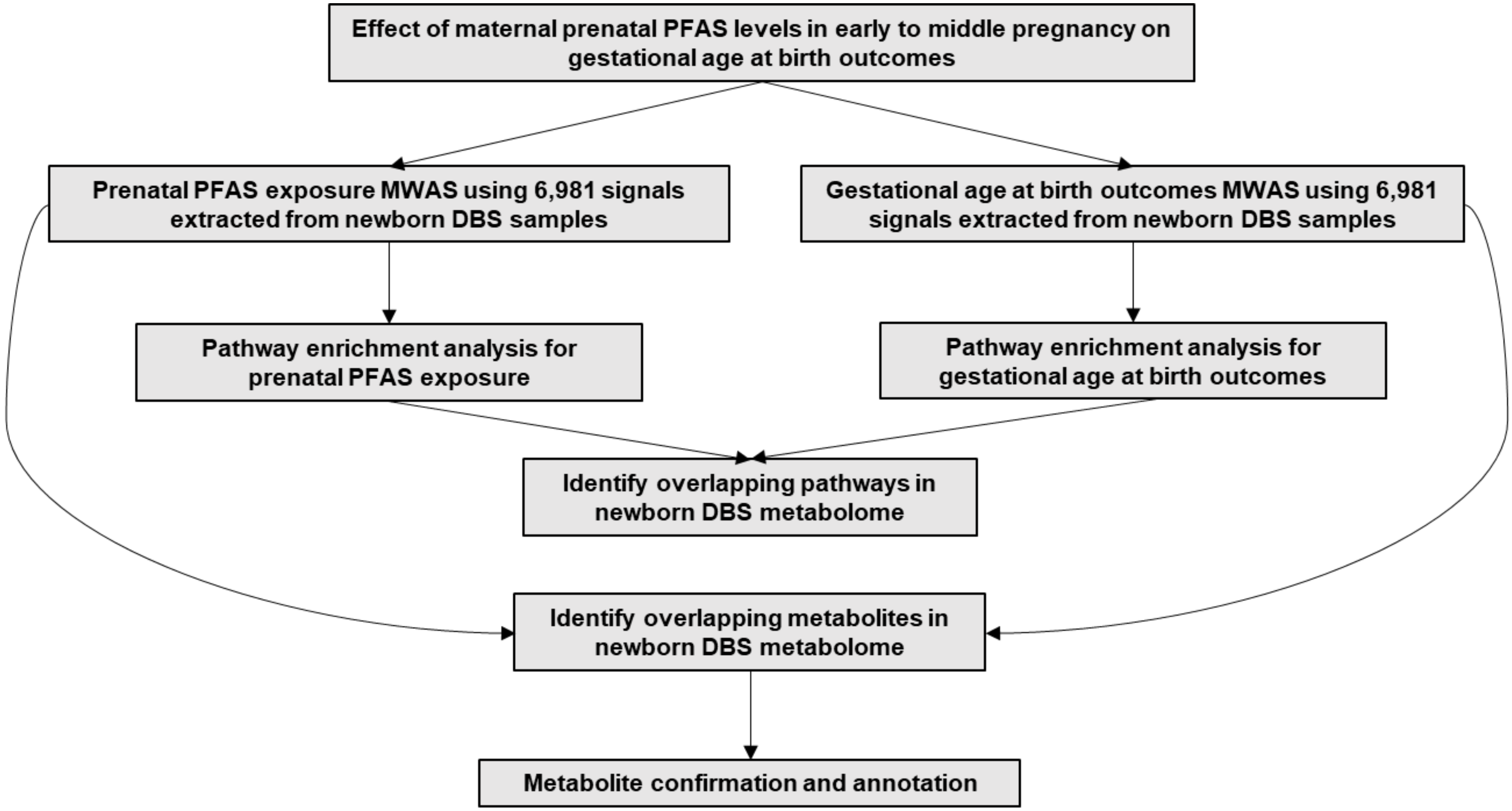

Abbreviations: PFAS = perfluoroalkyl substances; MWAS = metabolome-wide association study; DBS = dried blood spot.

Supplemental Table 1. Distribution and detection of serum PFAS concentrations (ng/mL) measured in 267 pregnant African American people in Atlanta, 2016 – 2020.

PFAS	LOD	Detection rate	GM (GSD)[a]	P25	P50	P75	Max.
PFOA	New York University:0.020<br>Wadsworth Center: 0.035<br>Emory University: 0.200	99%	0.57 (2.31)	0.42	0.63	0.96	3.42
PFNA	New York University: 0.027<br>Wadsworth Center: 0.020<br>Emory University: 0.100	98%	0.25 (2.26)	0.16	0.28	0.46	1.51
PFOS	New York University: 0.027<br>Wadsworth Center: 0.020<br>Emory University: 0.100	99%	1.43 (2.72)	1.04	1.64	2.46	9.59
PFHxS	New York University: 0.020<br>Wadsworth Center: 0.020<br>Emory University: 0.050	100%	1.09 (2.30)	0.66	1.07	1.93	6.17

Abbreviations: LOD = limits of detection; GM = geometric mean; GSD = geometric standard deviation; P25 = the 25th percentile; P50 = the 50th percentile; P75 = the 75th percentile; PFAS = perfluoroalkyl substances; PFOA = perfluorooctanoic acid; PFNA = perfluorononanoic acid; PFOS = perfluorooctane sulfonic acid; PFHxS = perfluorohexane sulfonic acid.

[a]The values below the LODs were replaced by LOD/$\sqrt{2}$.

Supplemental Table 2. Pearson correlations (ρ) between the $\log_2$-transformed serum PFAS concentrations (ng/mL) measured in 267 pregnant African American people in Atlanta, 2016 – 2020.

	**PFAS**			
	**PFOS**[a]	**PFOA**[a]	**PFNA**[a]	**PFHxS**[a]
**PFHxS**	0.23*	0.28*	0.42*	1
**PFNA**	0.42*	0.64*	1	
**PFOA**	0.57*	1		
**PFOS**	1			

Note: Pearson correlation tests were two-sided and performed with a significance level of p-value<0.05.

Abbreviations: PFAS = perfluoroalkyl substances; PFOA = perfluorooctanoic acid; PFNA = perfluorononanoic acid; PFOS = perfluorooctane sulfonic acid; PFHxS = perfluorohexane sulfonic acid.

[a] The values below the LODs were replaced by LOD/$\sqrt{2}$.

* p-value<0.05.

Supplemental Table 3. Associations between serum PFAS concentrations measured during early to middle pregnancy and gestational age at birth outcomes among African American mother-newborn dyads in Atlanta, 2016 – 2020.

	Gestational age at birth (weeks) [a] N = 267		Preterm birth [a,c] N = 149		Early term birth [a,c] N = 200		Spontaneous early birth [a,b,c] N = 200		Medically-indicated early birth [a,b,c] N = 149	
**PFAS (ng/mL)**	**β (95% CI)**	***p*-value**	**OR (95% CI)**	***p*-value**	**OR (95% CI)**	***p*-value**	**OR (95% CI)**	***p*-value**	**OR (95% CI)**	***p*-value**
**PFHxS**										
Per log_2-unit	-0.04 (-0.24,0.16)	0.67	1.03 (0.71,1.50)	0.87	0.92 (0.71,1.19)	0.51	0.86 (0.66,1.11)	0.24	1.19 (0.78,1.81)	0.41
Q1: <LOD – 0.66	*0 (referent)*	-	*1 (referent)*	-	*1 (referent)*	-	*1 (referent)*	-	*1 (referent)*	-
Q2: 0.66 – 1.07	-0.02 (-0.70,0.66)	0.95	3.65 (0.83,16.04)	0.09	1.80 (0.76,4.23)	0.18	1.44 (0.59,3.51)	0.42	6.39 (1.20,34.09)	0.03*
Q3: 1.07 – 1.93	-0.32 (-1.0,0.36)	0.36	2.76 (0.65,11.74)	0.17	1.41 (0.60,3.33)	0.43	1.18 (0.49,2.83)	0.71	5.22 (0.91,29.83)	0.06
Q4: 1.93 – 6.17	-0.10 (-0.77,0.58)	0.78	0.97 (0.21,4.47)	0.97	0.71 (0.29,1.74)	0.45	0.58 (0.24,1.45)	0.25	2.48 (0.40,15.23)	0.33
**PFOS**										
Per log_2-unit	-0.04 (-0.21,0.14)	0.68	1.20 (0.84,1.71)	0.32	1.16 (0.92,1.45)	0.21	1.12 (0.89,1.41)	0.32	1.26 (0.84,1.88)	0.27
Q1: <LOD – 1.04	*0 (referent)*	-	*1 (referent)*	-	*1 (referent)*	-	*1 (referent)*	-	*1 (referent)*	-
Q2: 1.04 – 1.64	0.61 (-0.05,1.28)	0.07	0.50 (0.13,1.90)	0.31	0.58 (0.25,1.38)	0.22	0.56 (0.23,1.33)	0.19	0.61 (0.15,2.44)	0.48
Q3: 1.64 – 2.46	0.17 (-0.50,0.84)	0.62	1.55 (0.41,5.90)	0.52	0.73 (0.31,1.74)	0.48	0.72 (0.29,1.75)	0.47	1.72 (0.47,6.26)	0.41
Q4: 2.46 – 9.59	-0.23 (-0.91,0.45)	0.51	1.27 (0.35,4.65)	0.72	1.89 (0.78,4.61)	0.16	1.41 (0.58,3.45)	0.45	2.29 (0.63,8.34)	0.21
**PFOA**										
Per log_2-unit	0.12 (-0.09,0.32)	0.27	0.92 (0.66,1.28)	0.60	1.59 (1.15,2.21)	0.01*	1.26 (0.96,1.67)	0.10	1.24 (0.81,1.88)	0.32
Q1: <LOD – 0.42	*0 (referent)*	-	*1 (referent)*	-	*1 (referent)*	-	*1 (referent)*	-	*1 (referent)*	-
Q2: 0.42 – 0.63	0.04 (-0.64,0.72)	0.90	0.89 (0.22,3.56)	0.87	2.85 (1.16,7.02)	0.02*	1.79 (0.73,4.40)	0.20	1.23 (0.31,4.87)	0.76
Q3: 0.63 – 0.96	0.31 (-0.36,0.99)	0.36	1.53 (0.41,5.70)	0.53	1.67 (0.66,4.21)	0.28	1.27 (0.50,3.19)	0.62	1.91 (0.50,7.32)	0.35
Q4: 0.96 – 3.42	0.30 (-0.39,0.99)	0.40	0.52 (0.12,2.29)	0.39	4.59 (1.78,11.89)	<0.001*	2.49 (0.98,6.31)	0.05	1.50 (0.38,5.97)	0.57
**PFNA**										
Per log_2-unit	0.08 (-0.12,0.29)	0.43	0.97 (0.67,1.41)	0.86	1.14 (0.87,1.50)	0.33	1.01 (0.78,1.30)	0.96	1.39 (0.88,2.20)	0.16
Q1: <LOD – 0.16	*0 (referent)*	-	*1 (referent)*	-	*1 (referent)*	-	*1 (referent)*	-	*1 (referent)*	-
Q2: 0.16 – 0.28	0.37 (-0.31,1.05)	0.28	0.39 (0.09,1.71)	0.21	0.91 (0.38,2.16)	0.83	1.01 (0.42,2.42)	0.98	0.49 (0.11,2.14)	0.34
Q3: 0.28 – 0.46	0.34 (-0.34,1.03)	0.33	0.39 (0.09,1.73)	0.22	1.12 (0.47,2.71)	0.80	1.14 (0.46,2.81)	0.77	1.57 (0.44,5.63)	0.49
Q4: 0.46 – 1.51	-0.17 (-0.86,0.52)	0.63	1.12 (0.32,3.94)	0.86	1.24 (0.52,3.00)	0.63	0.93 (0.38,2.28)	0.88	2.00 (0.56,7.13)	0.28

Note: Multivariable linear or logistic regression models were two-sided and performed with a significance level of p-value<0.05.

Abbreviations: OR, odds ratio; w, week; β, linear regression estimate coefficient; LOD, limit of detection; PFAS = perfluoroalkyl substances; PFHxS, perfluorohexane sulfonic acid; PFOS, perfluorooctane sulfonic acid; PFOA, perfluorooctanoic acid, PFNA, perfluorononanoic acid.

[a] Adjusted for maternal age, parity, education level, pre-pregnancy BMI, marijuana use, tobacco use, and neonatal sex.

[b] Early births include both PTB and ETB.

[c] Sample size shown was compared to 118 healthy full-term births in the early birth analyses.

* p-value<0.05.

Supplemental Table 4. Signals detected in 267 African American newborn dried blood spot samples and significantly associated with prenatal serum PFAS concentrations, 2016 – 2020.

PFAS	Bonferroni *q*-value<0.01	Bonferroni *q*-value<0.05	Bonferroni *q*-value<0.20	FDR *q*-value<0.05	FDR *q*-value<0.20	Raw *p*-value<0.05
PFOA	0	0	3	19	54	435
PFNA	154	242	361	1,676	2,694	2,400
PFOS	1	2	4	4	34	559
PFHxS	1,262	1,446	1,636	3,228	4,177	3,605

Abbreviations: FDR = Benjamini Hochberg procedure for false discovery rate correction of multiple comparisons; PFAS = perfluoroalkyl substances; PFOA = perfluorooctanoic acid; PFNA = perfluorononanoic acid; PFOS = perfluorooctane sulfonic acid; PFHxS = perfluorohexane sulfonic acid.

Supplemental Table 5. Signals detected in African American newborn dried blood spot samples and significantly associated with gestational age at birth outcomes, 2016 – 2020

**Birth outcome**	**N**	**Bonferroni *q*-value<0.01**	**Bonferroni *q*-value<0.05**	**Bonferroni *q*-value<0.20**	**FDR *q*-value<0.05**	**FDR *q*-value<0.20**	**Raw *p*-value<0.05**
Gestational age	267	142	173	220	745	1,495	1,594
Preterm birth	149	0	0	0	669	1,523	1,600
Early term birth	200	1	1	5	12	199	970
Spontaneous early birth[a]	200	3	7	15	244	1,266	1,463
Medically-indicated early birth[a]	149	2	5	13	78	318	803

Abbreviations: FDR = Benjamini Hochberg procedure for false discovery rate correction of multiple comparisons.

[a] Early births include both PTB and ETB.

Supplemental Table 6. Signals detected in 267 African American newborn dried blood spot samples and significantly associated with prenatal serum PFAS concentrations, 2016 – 2020: sensitivity analysis conducted without gestational week the maternal serum sample was collected.

**PFAS**	**Bonferroni *q*-value<0.01**	**Bonferroni *q*-value<0.05**	**Bonferroni *q*-value<0.20**	**FDR *q*-value<0.05**	**FDR *q*-value<0.20**	**Raw *p*-value<0.05**
PFOA	0	0	2	9	48	421
PFNA	157	243	363	1,677	2,698	2,404
PFOS	1	2	4	4	46	558
PFHxS	1,268	1,449	1,635	3,245	4,185	3,620

Abbreviations: FDR = Benjamini Hochberg procedure for false discovery rate correction of multiple comparisons; PFAS = perfluoroalkyl substances; PFOA = perfluorooctanoic acid; PFNA = perfluorononanoic acid; PFOS = perfluorooctane sulfonic acid; PFHxS = perfluorohexane sulfonic acid.

Supplemental Table 7. Overlapping signals across all MWAS performed at FDR q-value<0.05, except PFOA MWAS and PFOS MWAS p<0.05.

MWAS	PFOA	PFOS	PFNA	PFHxS
Gestational age	44	60	198	366
Preterm birth	41	56	178	322
Early term birth	0	0	5	6
Spontaneous early birth[a]	16	13	73	125
Medically-indicated early birth[a]	5	5	20	10

Abbreviations: FDR = Benjamini Hochberg procedure for false discovery rate correction of multiple comparisons; PFOA = perfluorooctanoic acid; PFNA = perfluorononanoic acid; PFOS = perfluorooctane sulfonic acid; PFHxS = perfluorohexane sulfonic acid.

[a] Early births include both PTB and ETB.

Supplemental Table 8. Newborn dried blood spot (DBS) biomarkers significantly associated with prenatal PFAS concentrations and gestational age at birth outcomes, by PFAS MWAS, in the Atlanta African American Maternal-Child cohort, 2016 – 2020.

Metabolite	m/z	RT (min)	Matching library ID	Class	PFAS MWAS							
					PFOA β	PFOA p-value	PFOS β	PFOS p-value	PFNA β	PFNA FDR q-value	PFHxS β	PFHxS FDR q-value
**OL1**												
N-Acetylleucine	173.1052	6.81	70912	Amino acids and proteins	-0.03	0.24	0.01	0.46	-0.03	0.33	-0.06	0.02
Hexanoyl glycine	173.1052	7.05	99463	Amino acids and proteins	-0.02	0.29	-0.01	0.76	-0.03	0.33	-0.07	0.003
Muricholic acid	373.2734	12.94	5283853	Bile acids	0.02	0.61	-0.05	0.22	0.07	0.27	0.13	0.01
7-Ketodeoxycholic acid	371.2577	13.11	188292	Bile acids	0.10	0.14	-0.02	0.70	0.09	0.35	0.16	0.03
3-Hydroxydodecanoyl carnitine	360.2741	11.69	71464535	Carnitines	0.03	0.51	0.01	0.71	0.04	0.56	0.12	0.03
6Z,9Z,12Z,15Z-Octadecatetraenoic acid	241.1949	15.57	5312508	Fatty acids and lipids	0.04	0.36	0.06	0.09	-0.02	0.78	-0.10	0.02
Linoleic acid	245.2261	16.54	5280450	Fatty acids and lipids	0.03	0.28	0.02	0.27	0.05	0.14	0.08	0.003
N-Acetylglucosamine	465.1690	0.74	24139	Redox reactions	0.03	0.64	0.02	0.73	0.12	0.15	0.29	3.1E-06
Monoisopropyl phthalate	231.0627	10.03	35118-50-4	Xenobiotics	0.30	0.003	0.18	0.047	0.26	0.05	-0.68	2.2E-10
**OL2a**												
Nordeoxycholic acid	378.2766	14.79	193905	Bile acids	-0.05	0.43	0.005	0.93	-0.06	0.48	-0.18	0.01
Tetradecenoyl-L-carnitine	387.3214	12.86	53481677	Carnitines	-0.05	0.56	-0.04	0.57	0.24	0.02	0.42	1.7E-06
L-DOPA	197.0687	1.44	6047	Chemical messengers	-0.05	0.04	0.01	0.55	-0.06	0.07	-0.05	0.06
Indoleacetaldehyde	192.1019	4.00	800	Chemical messengers	-0.002	0.92	-0.01	0.50	-0.06	0.04	-0.13	3.2E-10
Indole-3-methyl acetate	207.1127	9.58	74706	Chemical messengers	-0.09	0.03	-0.07	0.09	-0.14	0.01	-0.20	1.4E-05
2-Octenedioic acid	190.1074	7.33	6079438	Fatty acids and lipids	-0.07	0.08	0.01	0.76	-0.13	0.01	-0.20	3.4E-06
Benzoic acid	105.0335	7.47	243	Redox reactions	-0.02	0.46	-0.06	0.02	0.09	0.02	0.16	5.8E-07
4-Hydroxy-3-methoxycinnamaldehyde	196.0968	7.73	5280536	Redox reactions	0.01	0.56	-0.004	0.79	0.04	0.15	0.11	5.5E-09
Dextromethorphan	272.2003	8.73	5360696	Xenobiotics	0.05	0.35	-0.01	0.78	0.14	0.04	0.13	0.02
Piperonyl butoxide	339.2164	14.73	5794	Xenobiotics	-0.06	0.34	0.04	0.55	-0.21	0.02	-0.26	0.001
Fenuron	197.1284	7.15	7560	Xenobiotics	-0.03	0.37	0.003	0.90	-0.11	0.003	-0.22	8.1E-14
Ethylparaben	189.0522	9.23	HMDB32573	Xenobiotics	-0.11	0.66	-0.60	0.01	-0.08	0.85	-0.17	0.54
**OL2b**												
Glycylcholic acid	465.3090	11.78	23617285	Bile acids	-0.12	0.40	-0.31	0.02	0.001	0.996	0.04	0.84
Butanoylcarnitine	232.1542	7.13	213144	Carnitines	-0.06	0.20	0.02	0.65	-0.22	4.5E-05	-0.26	2.6E-08
N-Acetylserotonin	241.0949	7.81	903	Chemical messengers	-0.17	0.35	-0.36	0.01	0.03	0.93	0.10	0.65
4-Hydroxy-3-methoxyphenylglycol	184.0734	12.72	10805	Chemical messengers	0.01	0.62	-0.03	0.14	0.07	0.01	0.08	0.001
Stearidonic acid	259.2054	16.90	5312508	Fatty acids and lipids	-0.03	0.41	-0.02	0.62	-0.11	0.03	-0.13	0.002
Arachidonic acid	287.2366	16.90	444899	Fatty acids and lipids	0.04	0.43	0.01	0.89	0.21	4.1E-04	0.34	4.4E-11
3,4-Dimethoxyphenylpropanoic acid	249.0520	10.33	75019	Redox reactions	0.21	0.001	0.06	0.27	0.33	2.9E-05	-0.20	0.01
2-Phenylpropionic acid	151.0753	9.98	10296	Xenobiotics	0.01	0.54	-0.001	0.93	0.08	7.0E-04	0.07	4.1E-04
2,6-Dimethoxyphenol	172.0966	4.00	HMDB0034158	Xenobiotics	-0.003	0.90	-0.02	0.47	0.09	0.003	0.17	1.3E-11
9-Hydroxyfluorene	183.0803	5.80	74318	Xenobiotics	-0.03	0.59	-0.06	0.23	0.15	0.06	0.48	1.6E-15
**PDa**												
Trp Asp Leu	432.1994	10.61	16438	Amino acids and proteins	0.002	0.91	-0.02	0.19	0.06	0.003	0.07	9.6E-06
Gly Glu Tyr Ser Val	554.2459	12.74	264309	Amino acids and proteins	-0.02	0.85	0.01	0.86	0.18	0.09	0.22	0.01
Ile Glu	261.1444	2.46	0	Amino acids and proteins	-0.01	0.81	0.01	0.89	0.10	0.13	0.11	0.04
Asp Ile Lys	375.2236	2.61	17915	Amino acids and proteins	-0.01	0.75	-0.03	0.39	0.07	0.16	0.13	0.001
Ala Lys Val	316.2108	3.44	19862	Amino acids and proteins	-0.001	0.94	0.003	0.85	-0.03	0.20	-0.08	4.5E-05
Asp Ser Phe	368.1450	5.05	18491	Amino acids and proteins	0.003	0.96	-0.02	0.73	0.17	0.01	0.17	0.003
Pro Lys	244.1655	5.72	23869	Amino acids and proteins	-0.05	0.43	0.06	0.32	-0.37	9.8E-07	-0.50	1.3E-15
N-lactoyl-phenylalanine	238.1073	4.00	263655	Amino acids and proteins	0.06	0.04	0.03	0.20	-0.01	0.92	-0.17	4.6E-09
Asp Gly Ile	304.1501	4.70	21796	Amino acids and proteins	0.02	0.49	3.5E-05	0.999	0.10	0.002	0.12	2.0E-05
Isobutyryl carnitine	231.1469	7.40	34492	Carnitines	0.02	0.61	-0.02	0.53	-0.09	0.08	-0.19	5.3E-06
17-Hydroxyprogesterone	331.2265	10.68	44235	Chemical messengers	0.004	0.93	-0.001	0.98	0.08	0.26	0.16	0.002
4-Androsten-3,17-dione 19-aldehyde	301.1796	11.95	43199	Chemical messengers	-0.04	0.34	-0.01	0.88	-0.14	0.02	-0.09	0.10

15-Cyclohexyl pentanor prostaglandin F2-alpha	349.2370	10.49	58611-97-5	Chemical messengers	0.06	0.28	-0.03	0.47	0.11	0.17	0.18	0.004
9Z,11E,13E-Octadecatrienoic acid ethyl ester	306.2557	13.99	42021-86-3	Fatty acids and lipids	0.02	0.51	0.03	0.22	0.03	0.46	0.07	0.01
9,10-Dihydroxy-12Z-octadecenoic acid	315.2527	14.56	263399-34-4	Fatty acids and lipids	0.03	0.68	0.05	0.47	0.02	0.92	0.18	0.04
14(15)-EET methyl ester	317.2472	16.64	197508-63-7	Fatty acids and lipids	0.02	0.54	0.04	0.19	0.06	0.33	0.10	0.02
cis,cis-9,12-Octadecadien-1-ol	266.2606	16.67	506-43-4	Fatty acids and lipids	0.02	0.40	-0.01	0.69	0.04	0.10	0.06	0.004
O-Arachidonoylglycidol	361.2732	17.00	439146-24-4	Fatty acids and lipids	0.16	0.01	0.11	0.03	0.25	0.002	0.21	0.001
DMABA NHS ester	263.1024	4.45	96490	Redox reactions	-0.02	0.49	0.02	0.27	-0.10	0.002	-0.18	2.2E-12
beta-Nicotinamide adenine dinucleotide	332.5615	1.38	53-84-9	Redox reactions	-0.03	0.54	-0.08	0.03	0.07	0.30	0.21	4.7E-06
Pyridoxamine	169.0971	4.47	238	Redox reactions	-0.02	0.21	-0.02	0.16	-0.04	0.13	-0.05	0.01
S-Lactoylglutathione	380.1120	2.49	25138-66-3	Redox reactions	0.27	3.8E-04	0.14	0.03	0.17	0.08	-0.44	2.0E-08
S-(4-Nitrobenzyl)glutathione	443.1230	6.13	4098	Redox reactions	-0.03	0.40	-0.02	0.51	-0.02	0.75	0.11	0.003
Nandrolone	274.1930	12.84	434-22-0	Xenobiotics	-0.10	0.04997	0.02	0.60	-0.35	5.6E-09	-0.35	4.1E-12
3-Hydroxypyridine	96.0444	1.44	109-00-2	Xenobiotics	0.002	0.93	-0.02	0.29	0.07	0.01	0.08	3.1E-04

Note: Multivariable linear or logistic regression models were two-sided. No adjustments were made for multiple comparisons in the PFOA MWAS or PFOS MWAS. Abbreviations: m/z = mass-to-charge ratio; RT = retention time; FDR = Benjamini Hochberg procedure for false discovery rate correction of multiple comparisons; PFAS = perfluoroalkyl substances; MWAS = metabolome-wide association study; PFAS = perfluoroalkyl substances; PFOA = perfluorooctanoic acid; PFNA = perfluorononanoic acid; PFOS = perfluorooctane sulfonic acid; PFHxS = perfluorohexane sulfonic acid; OL1 = orthogonal level 1; OL2 = orthogonal level 2; PDa = public database orthogonal level.

Ontology levels: OL1, highly confident identification based on matching with in-house experimental standard library (IESL) via retention time (RT, with RT error≤|0.5|), exact mass (MS, with mass error<5ppm), and tandem mass similarity (MS/MS, with similarity ≥30); OL2a, confident identification based on matching with IPSL via MS and RT; OL2b, annotation for the isomer or derivatives of the compound listed but not the compound itself, based on matching with IPSL via MS and MS/MS; PDa, annotation based on matching with public database via MS and experimental MS/MS (could be the listed compound, or the isomer or derivative of the listed compound)

Supplemental Table 9. Newborn dried blood spot (DBS) biomarkers significantly associated with prenatal PFAS concentrations and gestational age at birth outcomes, by gestational age at birth outcomes MWAS, in the Atlanta African American Maternal-Child cohort, 2016 – 2020.

					Birth outcome MWAS									
**Metabolite**	**m/z**	**RT (min)**	**Matching library ID**	**Class**	**Gestational age β**	**Gestational age FDR q-value**	**PTB β**	**PTB FDR q-value**	**ETB β**	**ETB FDR q-value**	**Medically-indicated early birth[a] β**	**Medically-indicated early birth FDR q-value**	**Spontaneous early birth[a] β**	**Spontaneous early birth FDR q-value**
**OL1**														
N-Acetylleucine	173.1052	6.81	70912	Amino acids and proteins	-0.04	0.02	0.19	0.18	0.18	0.12	0.13	0.88	0.19	0.05
Hexanoyl glycine	173.1052	7.05	99463	Amino acids and proteins	-0.06	0.42	0.84	0.04	-0.12	0.77	0.61	0.02	0.03	0.95
Muricholic acid	373.2734	12.94	5283853	Bile acids	-0.07	0.002	0.51	0.01	0.17	0.32	0.08	0.83	0.20	0.16
7-Ketodeoxycholic acid	371.2577	13.11	188292	Bile acids	-0.15	3.7E-04	0.76	0.04	0.27	0.43	-0.59	0.25	0.27	0.34
3-Hydroxydodecanoyl carnitine	360.2741	11.69	71464535	Carnitines	0.002	0.97	0.14	0.50	0.17	0.26	0.24	0.50	0.25	0.02
6Z,9Z,12Z,15Z-Octadecatetraenoic acid	241.1949	15.57	5312508	Fatty acids and lipids	-0.08	0.04	0.54	0.07	0.24	0.41	-0.07	0.83	0.37	0.11
Linoleic acid	245.2261	16.54	5280450	Fatty acids and lipids	0.15	1.1E-07	-1.00	9.7E-06	-0.25	0.26	-0.43	0.19	-0.42	0.03
N-Acetylglucosamine	465.1690	0.74	24139	Redox reactions	0.16	7.0E-10	-0.94	8.4E-06	-0.32	0.15	NA	NA	-0.38	0.02
Monoisopropyl phthalate	231.0627	10.03	35118-50-4	Xenobiotics	-0.21	0.02	1.13	0.16	0.15	0.87	-0.33	0.61	0.07	0.95
**OL2a**														
Nordeoxycholic acid	378.2766	14.79	193905	Bile acids	0.11	0.01	-0.92	0.005	-0.01	0.99	0.78	0.29	-0.15	0.61
Tetradecenoyl-L-carnitine	387.3214	12.86	53481677	Carnitines	0.08	0.01	-0.45	0.06	0.05	0.86	-0.15	0.89	0.01	0.99
L-DOPA	197.0687	1.44	6047	Chemical messengers	-0.11	1.1E-05	0.82	5.5E-05	-0.03	0.91	-0.58	0.02	0.09	0.66
Indoleacetaldehyde	192.1019	4.00	800	Chemical messengers	0.05	0.39	-0.62	0.10	-0.38	0.24	-0.01	0.98	-0.50	0.05
Indole-3-methyl acetate	207.1127	9.58	74706	Chemical messengers	-0.09	1.6E-04	0.93	4.1E-08	0.05	0.84	0.05	0.98	0.26	0.09
2-Octenedioic acid	190.1074	7.33	6079438	Fatty acids and lipids	0.07	0.06	-0.43	0.06	-0.31	0.18	-0.11	0.56	-0.37	0.04
Benzoic acid	105.0335	7.47	243	Redox reactions	-0.16	0.002	1.04	0.02	0.15	0.69	-0.14	0.81	0.13	0.71
4-Hydroxy-3-methoxycinnamaldehyde	196.0968	7.73	5280536	Redox reactions	-0.12	0.03	0.10	0.85	0.03	0.96	-0.31	0.72	-0.08	0.82
Dextromethorphan	272.2003	8.73	5360696	Xenobiotics	0.03	0.64	-0.64	0.07	-0.12	0.76	-0.68	0.02	-0.24	0.42
Piperonyl butoxide	339.2164	14.73	5794	Xenobiotics	-0.05	0.06	0.50	0.01	0.19	0.34	0.01	0.98	0.23	0.14
Fenuron	197.1284	7.15	7560	Xenobiotics	0.12	0.03	-1.24	4.1E-04	-0.18	0.64	0.25	0.50	-0.38	0.20
Ethylparaben	189.0522	9.23	HMDB32573	Xenobiotics	-0.14	0.02	1.36	0.01	0.37	0.48	0.31	0.84	0.41	0.35
**OL2b**														
Glycylcholic acid	465.3090	11.78	23617285	Bile acids	0.18	0.01	-0.97	0.08	-0.41	0.42	-0.14	0.60	-0.62	0.12
Butanoylcarnitine	232.1542	7.13	213144	Carnitines	0.03	0.004	-0.06	0.55	-0.10	0.24	-0.11	0.85	-0.09	0.21
N-Acetylserotonin	241.0949	7.81	903	Chemical messengers	-0.01	0.87	-0.02	0.97	-0.38	0.22	0.00	0.997	-0.46	0.04
4-Hydroxy-3-methoxyphenylglycol	184.0734	12.72	10805	Chemical messengers	-0.05	0.03	0.41	0.003	0.17	0.25	0.15	0.73	0.23	0.04
Stearidonic acid	259.2054	16.90	5312508	Fatty acids and lipids	0.10	5.1E-05	-0.85	1.2E-06	-0.23	0.23	-0.53	0.45	-0.37	0.02
Arachidonic acid	287.2366	16.90	444899	Fatty acids and lipids	0.13	3.0E-06	-0.65	0.003	-0.33	0.12	-0.17	0.81	-0.41	0.02
3,4-Dimethoxyphenylpropanoic acid	249.0520	10.33	75019	Redox reactions	-0.04	0.03	0.27	0.07	-0.09	0.49	0.36	0.78	-0.08	0.51
2-Phenylpropionic acid	151.0753	9.98	10296	Xenobiotics	0.04	0.001	-0.18	0.04	-0.09	0.27	0.08	0.74	-0.07	0.34
2,6-Dimethoxyphenol	172.0966	4.00	HMDB0034158	Xenobiotics	-0.05	2.9E-04	0.29	0.01	0.11	0.30	-0.14	0.19	0.13	0.11
9-Hydroxyfluorene	183.0803	5.80	74318	Xenobiotics	-0.12	0.14	1.26	0.03	0.61	0.22	0.93	0.50	0.74	0.09
**PDa**														

Trp Asp Leu	432.1994	10.61	16438	Amino acids and proteins	-0.14	0.001	0.67	0.06	0.05	0.90	-0.05	0.89	0.12	0.70
Gly Glu Tyr Ser Val	554.2459	12.74	264309	Amino acids and proteins	0.17	1.7E-13	-0.95	2.7E-07	-0.30	0.12	-0.58	0.69	-0.40	0.01
Ile Glu	261.1444	2.46	0	Amino acids and proteins	-0.07	0.10	0.73	0.01	0.33	0.25	0.68	0.30	0.47	0.03
Asp Ile Lys	375.2236	2.61	17915	Amino acids and proteins	-0.16	0.02	0.82	0.19	0.06	0.94	-0.58	0.57	0.07	0.92
Ala Lys Val	316.2108	3.44	19862	Amino acids and proteins	0.15	1.2E-06	-0.82	0.001	-0.27	0.26	-3.28	0.78	-0.37	0.06
Asp Ser Phe	368.1450	5.05	18491	Amino acids and proteins	-0.08	0.03	0.68	0.01	0.20	0.47	0.50	0.57	0.31	0.13
Pro Lys	244.1655	5.72	23869	Amino acids and proteins	-0.08	0.01	0.46	0.07	0.06	0.80	0.21	0.55	0.04	0.87
N-lactoyl-phenylalanine	238.1073	4.00	263655	Amino acids and proteins	-0.13	0.18	0.23	0.79	0.12	0.87	0.37	0.02	0.41	0.42
Asp Gly Ile	304.1501	4.70	21796	Amino acids and proteins	-0.26	0.01	0.94	0.28	-0.14	0.88	-0.68	0.80	0.19	0.81
Isobutyryl carnitine	231.1469	7.40	34492	Carnitines	-0.15	0.02	0.71	0.18	0.48	0.31	-0.25	0.77	0.39	0.33
17-Hydroxyprogesterone	331.2265	10.68	44235	Chemical messengers	-0.49	0.06	4.41	0.049	-0.44	0.86	-0.18	0.68	1.31	0.40
4-Androsten-3,17-dione 19-aldehyde	301.1796	11.95	43199	Chemical messengers	0.04	0.35	0.09	0.75	-0.43	0.02	0.53	0.77	-0.40	0.02
15-Cyclohexyl pentanor prostaglandin F2-alpha	349.2370	10.49	58611-97-5	Chemical messengers	-0.19	0.002	1.06	0.03	0.29	0.56	0.00	0.995	0.28	0.55
9Z,11E,13E-Octadecatrienoic acid ethyl ester	306.2557	13.99	42021-86-3	Fatty acids and lipids	-0.12	0.001	0.52	0.11	0.09	0.79	-0.06	0.95	0.07	0.82
9,10-Dihydroxy-12Z-octadecenoic acid	315.2527	14.56	263399-34-4	Fatty acids and lipids	-0.08	0.01	0.26	0.37	0.25	0.26	0.02	0.95	0.20	0.28
14(15)-EET methyl ester	317.2472	16.64	197508-63-7	Fatty acids and lipids	0.04	0.18	-0.46	0.04	-0.23	0.26	-0.16	0.88	-0.37	0.02
cis,cis-9,12-Octadecadien-1-ol	266.2606	16.67	506-43-4	Fatty acids and lipids	-0.05	0.08	0.58	0.001	0.02	0.95	-0.18	0.84	0.25	0.11
O-Arachidonoylglycidol	361.2732	17.00	439146-24-4	Fatty acids and lipids	0.06	0.02	-0.32	0.12	-0.002	0.99	0.11	0.88	-0.01	0.97
DMABA NHS ester	263.1024	4.45	96490	Redox reactions	-0.21	0.04	1.28	0.24	0.37	0.68	0.13	0.37	0.56	0.45
beta-Nicotinamide adenine dinucleotide	332.5615	1.38	53-84-9	Redox reactions	0.09	0.01	-0.94	4.2E-04	-0.26	0.28	-0.07	0.77	-0.36	0.09
Pyridoxamine	169.0971	4.47	238	Redox reactions	0.20	4.1E-05	-1.93	7.3E-08	-0.22	0.55	-0.22	0.88	-0.53	0.08
S-Lactoylglutathione	380.1120	2.49	25138-66-3	Redox reactions	-0.27	0.01	0.95	0.30	-0.24	0.78	0.01	0.97	0.13	0.88
S-(4-Nitrobenzyl)glutathione	443.1230	6.13	4098	Redox reactions	0.23	4.0E-11	-1.31	1.2E-05	-0.27	0.37	0.38	0.64	-0.36	0.12
Nandrolone	274.1930	12.84	434-22-0	Xenobiotics	-0.04	0.06	0.42	0.002	0.04	0.79	-0.08	0.85	0.09	0.50
3-Hydroxypyridine	96.0444	1.44	109-00-2	Xenobiotics	-0.03	0.02	0.29	3.5E-03	0.09	0.42	-0.02	0.96	0.12	0.14

Note: Multivariable linear or logistic regression models were two-sided and adjusted for multiple comparisons with Benjamini-Hochberg procedure.

Abbreviations: m/z = mass-to-charge ratio; RT = retention time; FDR = Benjamini Hochberg procedure for false discovery rate correction of multiple comparisons; MWAS = metabolome-wide association study; PTB = preterm birth; ETB = early term birth; OL1 = orthogonal level 1; OL2 = orthogonal level 2; PDa = public database orthogonal level.

[a] Early births include both PTB and ETB.

Ontology levels: OL1, highly confident identification based on matching with in-house experimental standard library (IESL) via retention time (RT, with RT error≤|0.5|), exact mass (MS, with mass error<5ppm), and tandem mass similarity (MS/MS, with similarity ≥30); OL2a, confident identification based on matching with IPSL via MS and RT; OL2b, annotation for the isomer or derivatives of the compound listed but not the compound itself, based on matching with IPSL via MS and MS/MS; PDa, annotation based on matching with public database via MS and experimental MS/MS (could be the listed compound, or the isomer or derivative of the listed compound)

Supplemental Table 10. Overlapping pathways enriched in African American newborn dried blood spot samples and significantly associated with prenatal PFAS concentrations and gestational age at birth outcomes, 2016 – 2020.

Significance threshold	MWAS	Overlapping pathway	Overlap size	Pathway size	Overlap %	p-value
Raw p-value<0.05	PFOA	Alanine and aspartate metabolism	4	14	29%	0.033
		Arginine and proline metabolism	7	31	23%	0.046
		Aspartate and asparagine metabolism	12	56	21%	0.039
		Biopterin metabolism	4	14	29%	0.033
		Glycerophospholipid metabolism	9	32	28%	0.008
		Leukotriene metabolism	11	49	22%	0.029
		Lysine metabolism	6	23	26%	0.024
		Tryptophan metabolism	16	61	26%	0.005
		Urea cycle/amino group metabolism	10	38	26%	0.010
		Vitamin B3 (niacin) metabolism	6	16	38%	0.004
Raw p-value<0.05	PFOS	Drug metabolism - other enzymes	7	14	50%	0.002
		Glycerophospholipid metabolism	11	32	34%	0.005
		Histidine metabolism	5	15	33%	0.026
		Tryptophan metabolism	19	61	31%	0.005
		Tyrosine metabolism	22	80	28%	0.012
Bonferroni q-value<0.01	PFNA	Leukotriene metabolism	5	49	10%	0.005
		Lysine metabolism	3	23	13%	0.006
Bonferroni q-value<0.01	PFHxS	Biopterin metabolism	9	14	64%	0.007
		Cytochrome P450 metabolism	26	45	58%	0.003
		Drug metabolism - other enzymes	8	14	57%	0.022
		Tryptophan metabolism	36	61	59%	0.002
		Urea cycle/amino group metabolism	19	38	50%	0.025
		Vitamin B3 (niacin) metabolism	10	16	63%	0.007
FDR q-value<0.05	Preterm birth	Alanine and aspartate metabolism	5	14	36%	0.040
		Aspartate and asparagine metabolism	16	52	31%	0.032
		Cytochrome P450 metabolism	14	45	31%	0.032
		Lysine metabolism	8	23	35%	0.023
		Urea cycle/amino group metabolism	13	38	34%	0.014
FDR q-value<0.20	Early term birth	Alanine and aspartate metabolism	3	14	21%	0.008
		Arginine and proline metabolism	4	31	13%	0.013
		Aspartate and asparagine metabolism	6	56	11%	0.012
		Biopterin metabolism	4	14	29%	0.004
		Drug metabolism - other enzymes	3	14	21%	0.008
		Histidine metabolism	2	15	13%	0.049
		Tryptophan metabolism	6	61	10%	0.017
		Tyrosine metabolism	8	80	10%	0.011
		Urea cycle/amino group metabolism	5	38	13%	0.009
Bonferroni q-value<0.01	Spontaneous early birth[a]	Biopterin metabolism	3	14	21%	0.013
		Tryptophan metabolism	10	61	16%	0.003
		Urea cycle/amino group metabolism	5	38	13%	0.023

Bonferroni q-value<0.01	Medically-indicated early birth[a]	Glycerophospholipid metabolism	7	32	22%	0.010
		Leukotriene metabolism	10	49	20%	0.009
		Tryptophan metabolism	13	61	21%	0.004
		Urea cycle/amino group metabolism	7	38	18%	0.032

Note: The p-value is calculated based on permutations that randomly resamples the list of total features for a number of features equal to the significant set many times to create a γ null distribution.

Abbreviations: FDR = Benjamini Hochberg procedure for false discovery rate correction of multiple comparisons; PFAS = perfluoroalkyl substances; PFOA = perfluorooctanoic acid; PFNA = perfluorononanoic acid; PFOS = perfluorooctane sulfonic acid; PFHxS = perfluorohexane sulfonic acid.

[a] Early births include both PTB and ETB.

Supplemental Table 11. Pathways enriched in 267 African American newborn dried blood spot samples and significantly associated with prenatal serum PFAS concentrations, 2016 – 2020.

Significance threshold	MWAS	Pathway	Overlap size	Pathway size	Overlap %	p-value
Raw p-value<0.05	PFOA	Vitamin B3 (niacin) metabolism	6	16	38	0.004
		Tryptophan metabolism	16	61	26	0.005
		Vitamin E metabolism	10	35	29	0.006
		Glycerophospholipid metabolism	9	32	28	0.008
		Urea cycle/amino group metabolism	10	38	26	0.010
		N-Glycan degradation	3	8	38	0.023
		Lysine metabolism	6	23	26	0.024
		Leukotriene metabolism	11	49	22	0.029
		Alanine and aspartate metabolism	4	14	29	0.033
		Biopterin metabolism	4	14	29	0.033
		Aspartate and asparagine metabolism	12	56	21	0.039
		Nitrogen metabolism	2	4	50	0.041
		Vitamin H (biotin) metabolism	2	4	50	0.041
		Arginine and proline metabolism	7	31	23	0.046
Raw p-value<0.05	PFOS	Drug metabolism - other enzymes	7	14	50	0.002
		Tryptophan metabolism	19	61	31	0.005
		Glycerophospholipid metabolism	11	32	34	0.005
		Sialic acid metabolism	8	24	33	0.011
		Tyrosine metabolism	22	80	28	0.012
		Keratan sulfate degradation	3	6	50	0.018
		Pyrimidine metabolism	9	31	29	0.023
		Aminosugars metabolism	6	19	32	0.025
		Histidine metabolism	5	15	33	0.026
		Alkaloid biosynthesis II	3	7	43	0.029
		Linoleic acid metabolism	6	20	30	0.033
		Carnitine shuttle	6	21	29	0.044
		N-Glycan degradation	3	8	38	0.046
Bonferroni q-value<0.01	PFNA	Omega-3 fatty acid metabolism	2	6	33	0.003
		Vitamin E metabolism	4	35	11	0.005
		Leukotriene metabolism	5	49	10	0.005
		Lysine metabolism	3	23	13	0.006
Bonferroni q-value<0.01	PFHxS	Tryptophan metabolism	36	61	59	0.002
		Drug metabolism - cytochrome P450	26	45	58	0.003
		Vitamin B3 (niacin) metabolism	10	16	63	0.007
		Biopterin metabolism	9	14	64	0.007
		Carnitine shuttle	12	21	57	0.011
		Prostaglandin formation from dihomo gamma-linoleic acid	3	3	100	0.016
		Drug metabolism - other enzymes	8	14	57	0.022
		Urea cycle/amino group metabolism	19	38	50	0.025
		Linoleic acid metabolism	10	20	50	0.049

Note: The p-value is calculated based on permutations that randomly resamples the list of total features for a number of features equal to the significant set many times to create a γ null distribution.

Abbreviations: FDR = Benjamini Hochberg procedure for false discovery rate correction of multiple comparisons; PFAS = perfluoroalkyl substances; PFOA = perfluorooctanoic acid; PFNA = perfluorononanoic acid; PFOS = perfluorooctane sulfonic acid; PFHxS = perfluorohexane sulfonic acid.

Supplemental Table 12. Pathways enriched in African American newborn dried blood spot samples and significantly associated with gestational age at birth outcomes, 2016 – 2020.

Significance threshold	MWAS	Pathway	Overlap size	Pathway size	Overlap %	p-value
Bonferroni q-value<0.01	Gestational age	Vitamin B6 (pyridoxine) metabolism	2	6	33	6.04E-05
		Selenoamino acid metabolism	2	11	18	0.0004
		Glycine, serine, alanine and threonine metabolism	3	27	11	0.0004
		Fatty acid activation	2	12	17	0.0005
		Butyrate metabolism	2	14	14	0.0009
		Fatty acid activation	2	12	17	0.0005
FDR q-value<0.05	Preterm birth	Caffeine metabolism	8	11	73	0.001
		Urea cycle/amino group metabolism	13	38	34	0.014
		Lysine metabolism	8	23	35	0.023
		Vitamin B6 (pyridoxine) metabolism	3	6	50	0.028
		Cytochrome P450 metabolism	14	45	31	0.032
		Aspartate and asparagine metabolism	16	52	31	0.032
		Alanine and aspartate metabolism	5	14	36	0.040
FDR q-value<0.20	Early term birth	Biopterin metabolism	4	14	29	0.004
		Porphyrin metabolism	4	15	27	0.004
		Fatty acid activation	3	12	25	0.007
		Drug metabolism – other enzymes	3	14	21	0.008
		Alanine and aspartate metabolism	3	14	21	0.008
		Urea cycle/amino group metabolism	5	38	13	0.009
		Tyrosine metabolism	8	80	10	0.011
		Aspartate and asparagine metabolism	6	56	11	0.012
		Arginine and proline Metabolism	4	31	13	0.013
		Beta-Alanine metabolism	2	11	18	0.029
		Methionine and cysteine metabolism	3	26	12	0.029
		Glycine, serine, alanine and threonine metabolism	3	27	11	0.032
		Histidine metabolism	2	15	13	0.049
FDR q-value<0.05	Spontaneous early birth[a]	Tryptophan metabolism	10	61	16	0.003
		Biopterin metabolism	3	14	21	0.013
		Methionine and cysteine metabolism	4	26	15	0.019
		Urea cycle/amino group metabolism	5	38	13	0.023
FDR q-value<0.20	Medically-indicated early birth[a]	Vitamin B9 (folate) metabolism	5	13	38	0.002
		Tryptophan metabolism	13	61	21	0.004
		Leukotriene metabolism	10	49	20	0.009
		Glycerophospholipid metabolism	7	32	22	0.010
		Vitamin B3 (niacin) metabolism	4	16	25	0.016
		Urea cycle/amino group metabolism	7	38	18	0.032
		Hexose phosphorylation	3	13	23	0.044

Note: The p-value is calculated based on permutations that randomly resamples the list of total features for a number of features equal to the significant set many times to create a γ null distribution.

Abbreviations: FDR = Benjamini Hochberg procedure for false discovery rate correction of multiple comparisons.

[a] Early births include both PTB and ETB.

Supplemental Table 13. Pathways enriched in 267 African American newborn dried blood spot samples and significantly associated with prenatal PFAS concentrations, 2016 – 2020: sensitivity analysis conducted without gestational week the maternal serum sample was collected.

Significance threshold	MWAS	Pathway	Overlap size	Pathway size	Overlap %	p-value
Raw p-value<0.05	PFOA	Vitamin B3 (niacin) metabolism	6	16	38%	0.003
		Vitamin E metabolism	10	35	29%	0.004
		Urea cycle/amino group metabolism	10	38	26%	0.006
		Tryptophan metabolism	14	61	23%	0.009
		Glycerophospholipid metabolism	8	32	25%	0.012
		Leukotriene metabolism	11	49	22%	0.016
		Lysine metabolism	6	23	26%	0.016
		N-Glycan degradation	3	8	38%	0.018
		Alanine and aspartate metabolism	4	14	29%	0.024
		Biopterin metabolism	4	14	29%	0.024
		Nitrogen metabolism	2	4	50%	0.034
		Vitamin H (biotin) metabolism	2	4	50%	0.034
		Glutathione metabolism	3	10	30%	0.038
		Aspartate and asparagine metabolism	11	56	20%	0.046
Raw p-value<0.05	PFOS	Drug metabolism - other enzymes	7	14	50%	0.001
		Tryptophan metabolism	20	61	33%	0.001
		Glycerophospholipid metabolism	11	32	34%	0.002
		Tyrosine metabolism	22	80	28%	0.006
		Keratan sulfate degradation	3	6	50%	0.008
		Alanine and aspartate metabolism	5	14	36%	0.008
		Pyrimidine metabolism	9	31	29%	0.011
		Aminosugars metabolism	6	19	32%	0.012
		Histidine metabolism	5	15	33%	0.012
		Linoleic acid metabolism	6	20	30%	0.016
		Carnitine shuttle	6	21	29%	0.022
		N-Glycan degradation	3	8	38%	0.023
		Vitamin E metabolism	9	35	26%	0.029
		Nitrogen metabolism	2	4	50%	0.034
		Fructose and mannose metabolism	3	9	33%	0.037
		Ascorbate (Vitamin C) Metabolism	5	19	26%	0.046
Bonferroni q-value<0.01	PFNA	Omega-3 fatty acid metabolism	2	6	33%	0.003
		Vitamin E metabolism	4	35	11%	0.005
		Leukotriene metabolism	5	49	10%	0.005
		Carnitine shuttle	2	21	10%	0.038
Bonferroni q-value<0.01	PFHxS	Biopterin metabolism	11	14	79%	0.002
		Tryptophan metabolism	36	61	59%	0.002
		Cytochrome P450 metabolism	26	45	58%	0.004
		Drug metabolism – other enzymes	9	14	64%	0.008
		Vitamin B3 (niacin) metabolism	10	16	63%	0.008
		Carnitine shuttle	12	21	57%	0.013
		Urea cycle/amino group metabolism	20	38	53%	0.015
		Prostaglandin formation from dihomo gamma-linoleic acid	3	3	100%	0.018

Note: The p-value is calculated based on permutations that randomly resamples the list of total features for a number of features equal to the significant set many times to create a γ null distribution.
Abbreviations: FDR = Benjamini Hochberg procedure for false discovery rate correction of multiple comparisons; PFAS = perfluoroalkyl substances; PFOA = perfluorooctanoic acid; PFNA = perfluorononanoic acid; PFOS = perfluorooctane sulfonic acid; PFHxS = perfluorohexane sulfonic acid.
