# Supplementary material for: Newborn metabolomic signatures of maternal per- and polyfluoroalkyl substance exposure and reduced length of gestation
Source: Nat Commun. 2023 May 30;14:3120. doi: 10.1038/s41467-023-38710-3 (PMC10229585; doi:10.1038/s41467-023-38710-3)
Supplement: Supplementary file 2 — Reporting Summary [file 41467_2023_38710_MOESM2_ESM.pdf]

nature portfolio

Corresponding author(s): Donghai Liang
Last updated by author(s): May 10, 2023

Reporting Summary

Nature Portfolio wishes to improve the reproducibility of the work that we publish. This form provides structure for consistency and transparency in reporting. For further information on Nature Portfolio policies, see our Editorial Policies and the Editorial Policy Checklist.

Statistics

For all statistical analyses, confirm that the following items are present in the figure legend, table legend, main text, or Methods section.

n/a	Confirmed	
☐	☒	The exact sample size (*n*) for each experimental group/condition, given as a discrete number and unit of measurement
☐	☒	A statement on whether measurements were taken from distinct samples or whether the same sample was measured repeatedly
☐	☒	The statistical test(s) used AND whether they are one- or two-sided<br>*Only common tests should be described solely by name; describe more complex techniques in the Methods section.*
☐	☒	A description of all covariates tested
☐	☒	A description of any assumptions or corrections, such as tests of normality and adjustment for multiple comparisons
☐	☒	A full description of the statistical parameters including central tendency (e.g. means) or other basic estimates (e.g. regression coefficient) AND variation (e.g. standard deviation) or associated estimates of uncertainty (e.g. confidence intervals)
☐	☒	For null hypothesis testing, the test statistic (e.g. *F*, *t*, *r*) with confidence intervals, effect sizes, degrees of freedom and *P* value noted<br>*Give P values as exact values whenever suitable.*
☒	☐	For Bayesian analysis, information on the choice of priors and Markov chain Monte Carlo settings
☒	☐	For hierarchical and complex designs, identification of the appropriate level for tests and full reporting of outcomes
☐	☒	Estimates of effect sizes (e.g. Cohen's *d*, Pearson's *r*), indicating how they were calculated

Our web collection on statistics for biologists contains articles on many of the points above.

Software and code

Policy information about availability of computer code

Data collection	All data collection were done on password-protected and encrypted tablet computers with REDCap software, which is a software that allows for the encrypted transfer of data to the Emory server over the internet in a HIPAA-compliant, encrypted manner.
Data analysis	All statistical analyses were conducted in R (Boston, MA, USA, Version 4.1.0). The UHPLC-HR-MS data were processed by Progenesis QI (version 2.1, Waters Corporation) for peak identification and alignment. Pathway enrichment analyses were performed using the bioinformatics software Mummichog (Version 1.0.10), which predicts biological networks, pathways, and metabolites based on significant signals with tentative chemical identities.

For manuscripts utilizing custom algorithms or software that are central to the research but not yet described in published literature, software must be made available to editors and reviewers. We strongly encourage code deposition in a community repository (e.g. GitHub). See the Nature Portfolio guidelines for submitting code & software for further information.

Data

Policy information about availability of data

All manuscripts must include a data availability statement. This statement should provide the following information, where applicable:

- Accession codes, unique identifiers, or web links for publicly available datasets
- A description of any restrictions on data availability
- For clinical datasets or third party data, please ensure that the statement adheres to our policy

The raw and processed metabolomics data generated in this study have been deposited in the Metabolomics Workbench (https://

www.metabolomicsworkbench.org/ Study ID ST002692) by the UNC Human Health Exposure Analysis Resource (HHEAR) Laboratory. The clinical outcome and PFAS exposure data are available under restricted access to protect the privacy of the study participants, access can be obtained by emailing corresponding authors Drs. Liang and Dunlop. Requests will be responded within 10 business days. The demographic covariates data are protected and are not available due to data privacy laws. All the source data for figures and tables, coding materials, and data protocols are provided in the Supplementary Information/Source Data file.

Source data are provided with this paper.

Human research participants

Policy information about studies involving human research participants and Sex and Gender in Research.

Reporting on sex and gender

There were 139 (52%) newborns assigned female sex at birth. Neonatal sex was believed to affect the exposures and outcomes, and thus adjusted in the models in all analyses.

Population characteristics

The characteristics of 267 African American pregnant people and newborns included in our study are summarized in Table 1. Prior to pregnancy, the majority of mothers had a BMI considered overweight (n=58; 22%) or with obesity (n=109; 41%), were parous (n=155; 58%), and did not use tobacco (n=239; 90%) or marijuana (n=177; 66%). At enrollment, the average participant age was 25.6 years (SD=5.2) and 163 (61%) of the mothers were in the first trimester. Participants predominantly had a high school education or less (n=153; 57%), public health insurance with Medicaid (n=218; 82%), and an income level 132% or lower times that of the Federal Poverty Level (n=153; 57%).

There were 139 (52%) newborns assigned female sex at birth. The average gestational age at delivery was 38.7 weeks (SD=2.0), with a total of 118 (51%) healthy and full-term, 31 (12%) preterm, and 82 (31%) early term. Among the early births (PTB or ETB) prior to full-term, 82 (69%) followed spontaneous labor and 31 (26%) followed medically-indicated induction or C-section (Figure S2).

Recruitment

Participants from the Atlanta African American Maternal-Child Cohort (ATL AA hereafter) were included in the present analysis. This ongoing, prospective birth cohort enrolls pregnant African Americans between 6–17 weeks gestation at Emory Midtown Hospital and Grady Hospital, which serve socioeconomically diverse populations in Atlanta, Georgia, and extends dyad follow-up through age six. Additional information regarding the cohort profile and data collection is described in detail elsewhere. Participants were eligible for inclusion if they self-identified as African American or Black, and were born in the US, between 18–40 years old, pregnant with a singleton, fluent in English, and had no chronic medical conditions. Participant data are confidential and proprietary information to the ATL AA cohort. All participants were recruited to participate in this study from the prenatal care clinics of two metropolitan hospitals in Atlanta, GA, affiliated with Emory University Woodruff Health Sciences Center: Grady Memorial Hospital, a county-supported hospital that serves as a safety net for low-income patients; and Emory University Hospital Midtown, a private hospital that serves patients from a wide economic range. There is no obvious self-selection bias or bias beyond those related to the potential difference among participants who wished to consent to participate in the study compared to those who did not, which is common in all prospective cohort study.

Ethics oversight

All participants provided written, informed consent to participate in the study, which was approved by the Institutional Review Board at Emory University (approval reference number 68441).

Note that full information on the approval of the study protocol must also be provided in the manuscript.

Field-specific reporting

Please select the one below that is the best fit for your research. If you are not sure, read the appropriate sections before making your selection.

☐ Life sciences ☐ Behavioural & social sciences ☒ Ecological, evolutionary & environmental sciences

For a reference copy of the document with all sections, see nature.com/documents/nr-reporting-summary-flat.pdf

Ecological, evolutionary & environmental sciences study design

All studies must disclose on these points even when the disclosure is negative.

Study description

In a prospective birth cohort, we sought to profile the neonatal metabolome for molecular signatures of maternal PFAS concentrations during early to middle pregnancy and gestational age at birth outcomes among African American mother-newborn dyads in Atlanta, Georgia. Based on prior work, we hypothesized that prenatal PFAS exposure interferes with gestational length and fetal growth. Additionally, we analyzed newborn dried blood spots (DBS), a minimally invasive biospecimen used for screening within 48 hours of birth, with high-resolution metabolomics and the MITM framework to identify and measure the underlying metabolites and pathways.

Research sample

The present study was designed to investigate and elucidate the molecular connection between maternal exposures to PFAS, newborn metabolic perturbations, and fetal growth restriction among African American pregnant people, as they disproportionately suffer from a range of environmental exposures and adverse birth outcomes, compared to U.S. women of other races/ethnicities. As such, all participants in this study were participants enrolled in the Atlanta African American Maternal-Child (ATL AA) Cohort who self-identify as African American or Black. This ongoing, prospective birth cohort enrolls pregnant African Americans between 6–17

	weeks gestation at Emory Midtown Hospital and Grady Hospital, which serve socioeconomically diverse populations in Atlanta, Georgia, and extends dyad follow-up through age six. The ATL AA cohort is generally representative of the socioeconomically diverse African American populations in Atlanta, Georgia. A criterion for inclusion in this cohort, is that the mother self-identifies as African American (i.e., a US-born Black woman) and between 18- and 40-years of age, who had previously enrolled in our Prenatal Cohort study and who gave permission for their data and biosamples to be used for environmental chemical assessments and omics profiling.
Sampling strategy	A total of 279 mothers and newborns enrolled in the ATL AA cohort had serum and DBS samples, respectively. At the time of our analysis, there were 273 participants with complete data available for PFAS measurements and DBS metabolomics. The live births occurred between June 2016 and June 2020. We excluded newborns with congenital anomalies (n=6) from our analysis. The remaining 267 dyads were included in the PFAS metabolome-wide association studies (MWAS), gestational age MWAS, and prenatal PFAS-gestational age analyses.
Data collection	Sociodemographic and gestational age at birth outcomes were collected from medical record abstraction by medical personnel. All data collection were done by the ATL AA Cohort study team led by Dr. Anne Dunlop on password-protected and encrypted tablet computers with REDCap software, which is a software that allows for the encrypted transfer of data to the Emory server over the internet in a HIPAA-compliant, encrypted manner. The participants contributed blood samples in the clinical visits, which were collected by the same study team and stored at the -80 freezer. Newborn DBS samples are routinely collected at time of birth for medical screening and public health surveillance by the Georgia Department of Public Health then archived for future biomonitoring purposes.
Timing and spatial scale	The live births occurred between June 2016 and June 2020. Maternal samples were collected between gestation weeks 6-17; while the dried blood spots were collected from the newborn within 48 hours of their births. All the study participants lived in the metropolitan area in the city of Atlanta, Georgia, USA.
Data exclusions	We excluded newborns with congenital anomalies (n=6) from our analysis.
Reproducibility	This is a prospective observational study without experiment.
Randomization	Not applicable as there is no group assigned for exposure/intervention. For the metabolomics analysis and the targeted exposure assessment on PFAS, study samples were randomized before sample preparation and data acquisition.
Blinding	All biological samples were blinded to the technician who performed the targeted exposure assessment and untargeted metabolomics profiling.

Did the study involve field work? ☐ Yes ☒ No

Reporting for specific materials, systems and methods

We require information from authors about some types of materials, experimental systems and methods used in many studies. Here, indicate whether each material, system or method listed is relevant to your study. If you are not sure if a list item applies to your research, read the appropriate section before selecting a response.

Materials & experimental systems

n/a	Involved in the study	
☒	☐	Antibodies
☒	☐	Eukaryotic cell lines
☒	☐	Palaeontology and archaeology
☒	☐	Animals and other organisms
☒	☐	Clinical data
☒	☐	Dual use research of concern

Methods

n/a	Involved in the study	
☒	☐	ChIP-seq
☒	☐	Flow cytometry
☒	☐	MRI-based neuroimaging
